# Supplementary material for: Membrane Tension Gates ERK-Mediated Regulation of Pluripotent Cell Fate
Source: Cell Stem Cell. 2021 Feb 4;28(2):273–284.e6. doi: 10.1016/j.stem.2020.10.018 (PMC7875115; doi:10.1016/j.stem.2020.10.018)
Supplement: Document S1. Figures S1–S6 [file mmc1.pdf]

Cell Stem Cell, Volume *28*

Supplemental Information

Membrane Tension Gates ERK-Mediated Regulation of Pluripotent Cell Fate

Henry De Belly, Aki Stubb, Ayaka Yanagida, Céline Labouesse, Philip H. Jones, Ewa K. Paluch, and Kevin J. Chalut

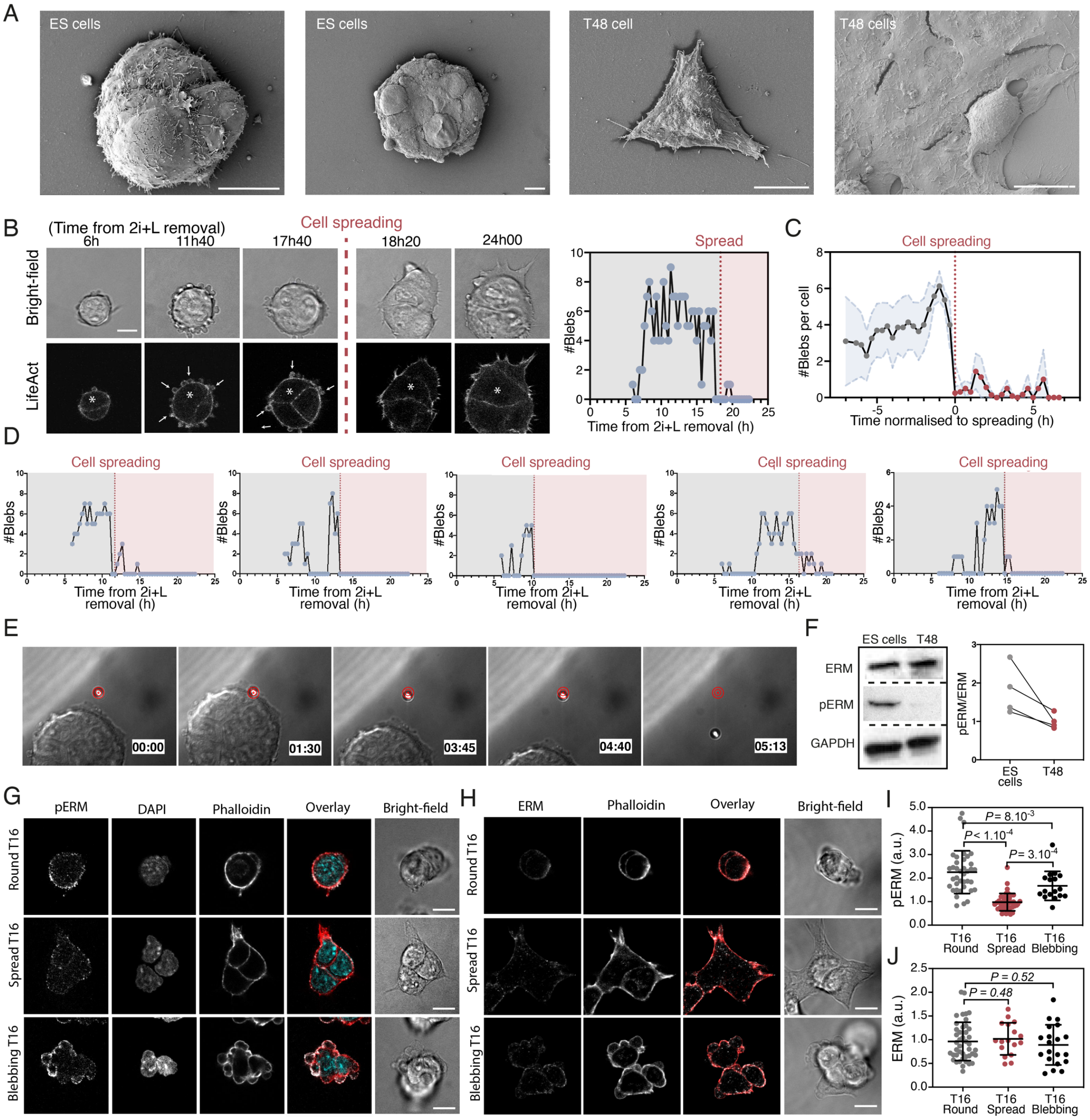

Figure S1: Membrane to cortex attachment and membrane tension are decreased during early differentiation (related to Figure 1). A, Representative SEM images of ES and T48 cells. Scale bars, 10 µm. **B,** Left: time-lapse of representative ES cells expressing LifeAct-GFP and exiting naïve pluripotency. White arrows: cellular blebs. Red dotted line indicates approximate time of cell spreading. Right: quantification of the number of blebs in the cell marked with a white star. **C,** Quantification of cell blebbing during exit from naïve pluripotency. Time is normalised to the time of spreading (mean ± SD, n = 20, N=2). **D**, Quantification of blebbing in six different ES cells expressing LifeAct-GFP during exiting naïve pluripotency. Red dotted line indicates approximate time of cell spreading. Bleb numbers per cell significantly decrease once the cell is spread (pink shaded area). **E**, Time lapse of a membrane tension measurement in a colony of ES cell. Time is in minutes:seconds. A red target is placed at the centre of the trap to help visualize bead displacement. **F**, Representative western blot for ERM, pERM and GAPDH in ES

cells and T24 cells and corresponding quantification (N=4, lines connect data points from individual experiments) **G**, Images of a single z-plane in fixed T16 cells. Three different cell morphologies are observed at T16: round, spread and blebbing. Cells were immunostained for pERM, and stained with DAPI and phalloidin. **H**, Similar as D with ERM instead of pERM. **I, J**, Estimated pERM and ERM and density in round, spread and blebbing T16 cells. Protein density was estimated from the mean intensity of sum z projections, corrected for background, and normalised to the mean of T16 round cells. (Means ± SD, N=3). For all panels, unless otherwise indicated, p-values established by Welch's unpaired student t-test.

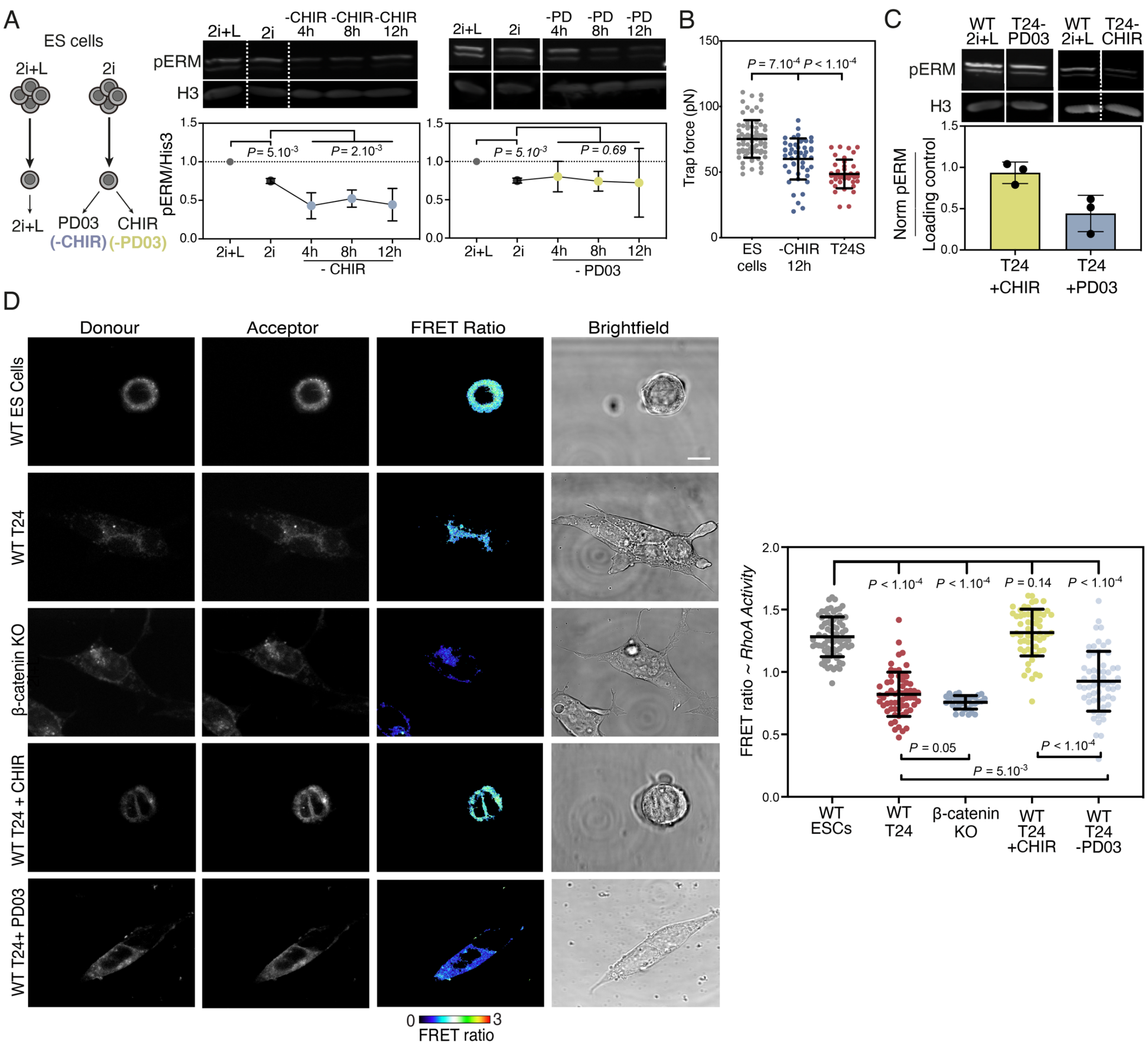

Figure S2: pERM activity and membrane tension during early differentiation are regulated by β-catenin and RhoA (related to Figure 2). A, Fluorescent Western Blot and associated quantification for pERM and Histone 3 (his 3) of cells cultured in 2i+L or from 2i and replated with PD03 or CHIR only for various timepoints (N=5). *P* values are calculated using a two-way analysis of variance (ANOVA), using experiment repeats and medium condition as variables, and indicated in the figure. **B**, Trap force measurements of WT ES, T24S (from Figure 1E) and cells cultured in medium lacking Chir (from 2i) (N=3). **C,** Top, representative fluorescent western blots for phospho-ERM and His3 of ES cells and T24 cells cultured in differentiation medium from 2i+L with either CHIR (annotated -PD03) or PD03 (annotated -CHIR). Bottom, associated quantification (N=3). **D**, Left, representative images of WT ES, T24, β-catenin KO cells and cells from 2i+L cultured for 24h in differentiation medium (N2B27) containing either PD03 (annotated -CHIR) or CHIR (annotated -PD03). Right, associated quantification (N=3). Graphical data represents mean ± SD. *P* values are calculated by Welch's unpaired student t-test (unless otherwise indicated). Scale bars = 10μm.

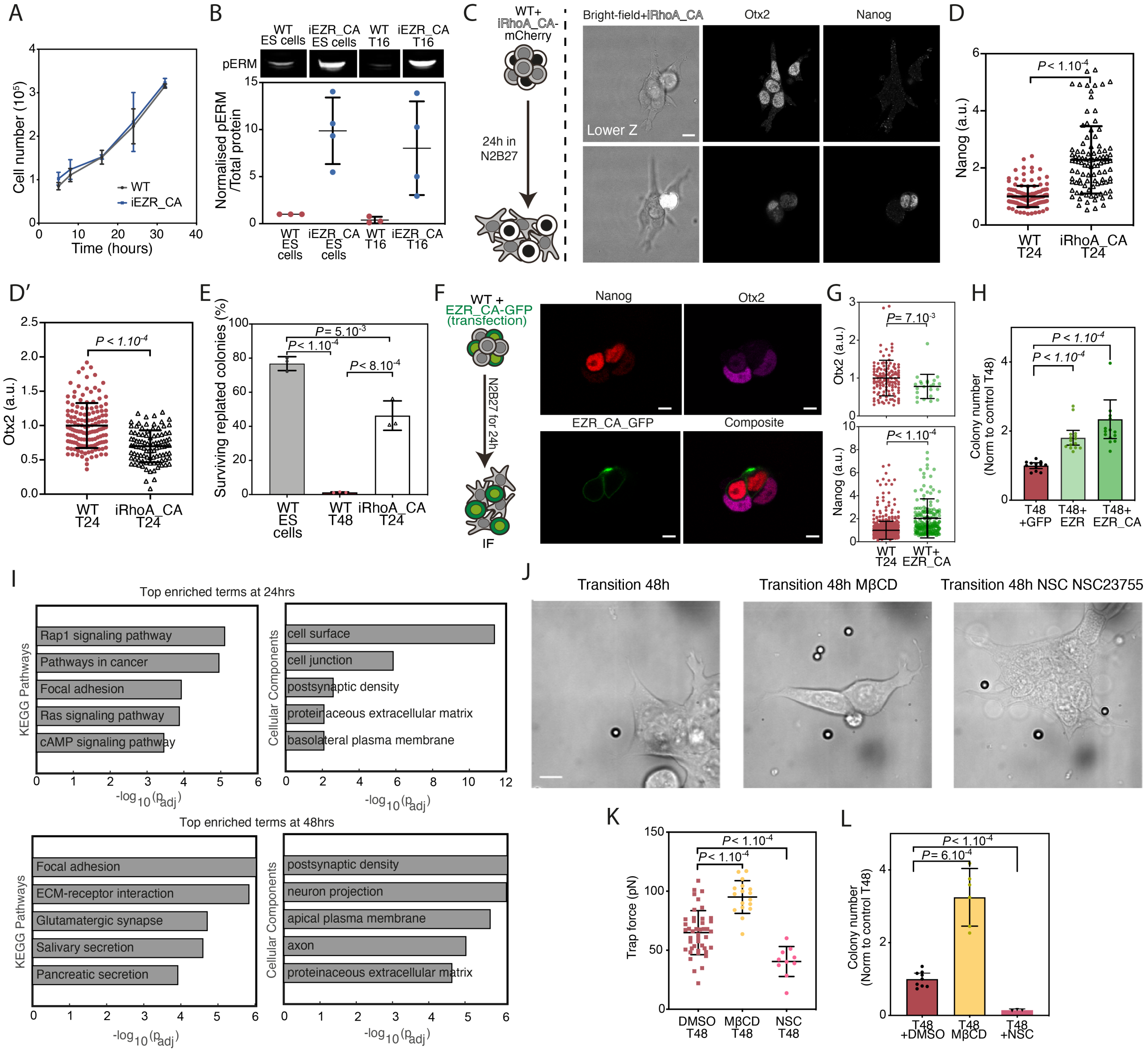

Figure S3: Increasing membrane tension inhibits early differentiation (related to Figure 3). A, Proliferation curves of WT and iEZR_CA ES cells (N=3). **B**, Quantification of phospho-ERM normalised to total protein levels in WT and iEZR_CA ES and T16 cells. Data are normalised to the mean of WT ES cells (N=4). **C,** Representative images of two z plane of a mix of WT and iRhoA_CA T24 cells immunostained for Otx2 and Nanog. **D,** Quantification of Nanog and Otx2 expression in WT and iRhoA_CA T24 cells, normalised to WT mean levels (N=3). **E**, Left, schematic of clonogenicity assay used as a measure of naïve pluripotency. Right: quantification of the percentage of surviving replated cells in a clonogenicity assay using WT and iRhoA_CA cells. Cells replated directly from 2i+L are used as a positive control (N=3). **F**, Representative single z-planes of a mix of control and EZR_CA transfected (positive for the EZR_CA _GFP) T24 cells immunostained for Otx2 and Nanog. **G**, Quantification of Nanog and Otx2 expression in T24 cells transfected or not with EZR_CA_GFP, normalised to T24 control mean

(N=2). **H,** Quantification of surviving replated cells in a clonogenicity assay using T48 cells transfected with either GFP (control), EZR, EZR_CA. (N=4) **I,** Top 5 KEGG pathways terms and Cellular Components terms that are enriched in the differentially regulated genes (n = 1165 genes at T24 and n = 1922 genes at T48). A threshold of > 5 was used for mean log2 expression, of >= 2.0 for fold enrichment, and of > 1% for proportion of regulated in each annotation. Adjusted p-value threshold was set at 0.1. The enriched annotations were sorted on increasing p-value and the top 5 were selected. **J**, Representative images of T48 cells treated with DMSO, NSC23766 or MβCD. MβCD was used at 1 mM for 1 h every 24 h. NSC23766 was used at 25 μM for 1 h every 24 h (N=3). **K**, Trap force measurements of T48 cells treated with DMSO, NSC23766 or MβCD at similar concentrations as in J. (N=2). **L**, Clonogenicity assay used to measure efficiency of exit from naïve pluripotency using T48 cells and different treatments to affect membrane tension. T48 cells replated from N2B27+DMSO were used as control for drug treatment. (N=3). Graphical data represents mean ± SD unless specified otherwise. Each condition is normalised to their respective control. *P* values are calculated by Welch's unpaired student t-test. Scale bars = 10μm.

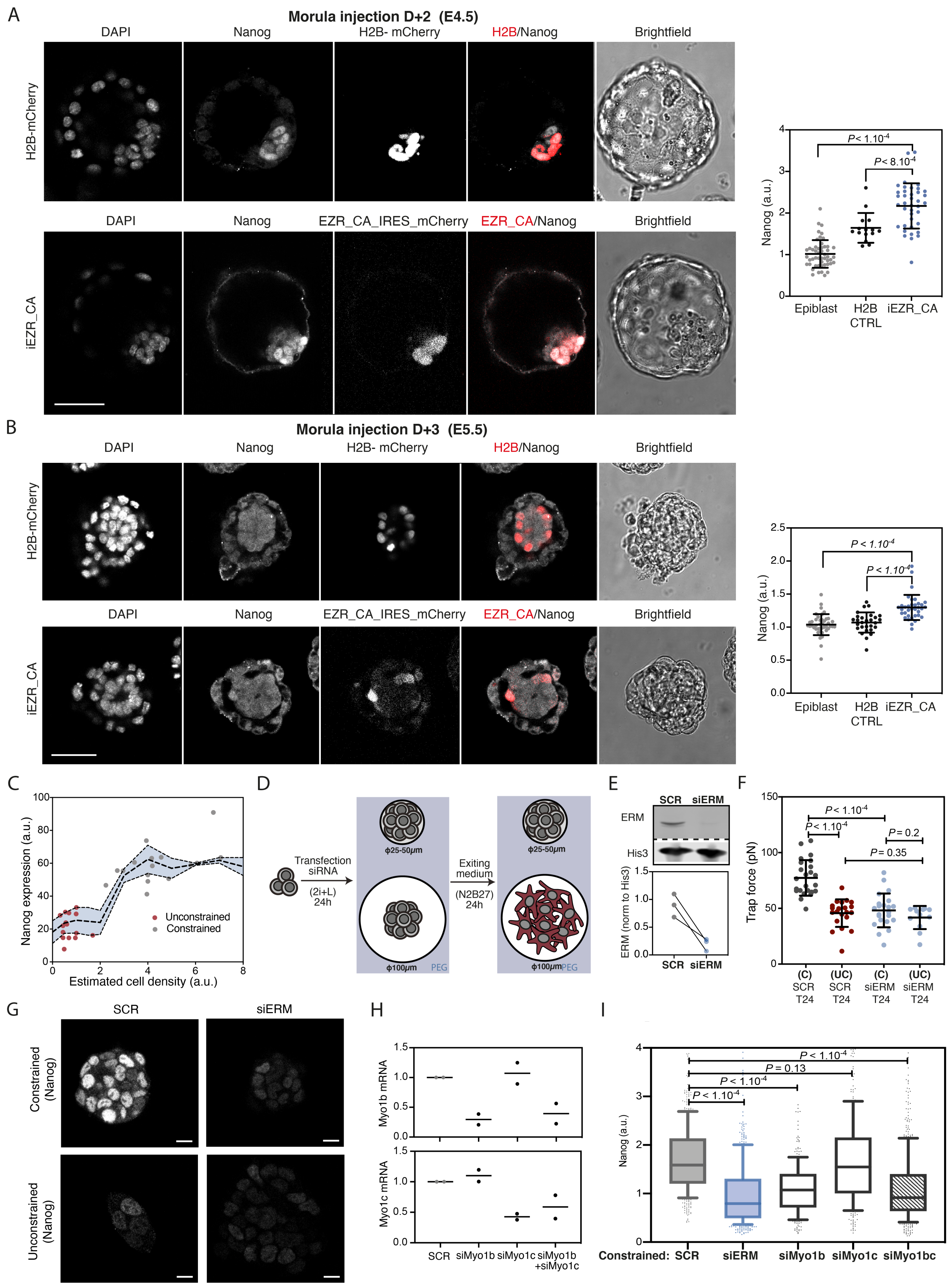

A
Morula injection D+2 (E4.5)
DAPI
Nanog
H2B- mCherry
H2B/Nanog
Brightfield
H2B-mCherry
EZR_CA_IRES_mCherry
EZR_CA/Nanog
iEZR_CA
Nanog (a.u.)
P< 1.10-4
P< 8.10-4
Epiblast
H2B CTRL
iEZR_CA
B
Morula injection D+3 (E5.5)
P < 1.10-4
C
Nanog expression (a.u.)
Estimated cell density (a.u.)
Unconstrained
Constrained
D
Transfection siRNA
(2i+L) 24h
ϕ25-50μm
ϕ100μm
PEG
Exiting medium
(N2B27) 24h
E
SCR
siERM
ERM
His3
ERM (norm to His3)
F
Trap force (pN)
P< 1.10-4
P= 0.2
P= 0.35
(C) SCR T24
(UC) SCR T24
(C) siERM T24
(UC) siERM T24
G
SCR
siERM
Constrained (Nanog)
Unconstrained (Nanog)
H
Myo1b mRNA
Myo1c mRNA
SCR
siMyo1b
siMyo1c
siMyo1b +siMyo1c
I
Nanog (a.u.)
P < 1.10-4
P = 0.13
Constrained: SCR
siERM
siMyo1b
siMyo1c
siMyo1bc

Figure S4: Maintaining a high membrane tension impairs exit from naïve pluripotency independently of cell shape (related to Figure 4). A, B, Representative images and associated quantification of morula injection experiment in which either control cells (H2B-mCherry) or iEZR_CA were injected at the 8 cells stage into the mouse embryo, which was then maintained in culture for 48 or 72h (see methods for details) (N=2). **C**, Same Nanog expression as in Figure 4C plotted in function of estimated cell density. Cell density was estimated by counting the number of cells and dividing by the pattern area. Data are colored based on size of pattern (red for 100 μm diameter and grey for 25-50 μm diameter). **D**, Schematic of the micropatterning assay combined with transfection of plasmids or siRNA. **E**, Fluorescent Western blot (inverted contrast) for ERM and Histone 3 in cells transfected with siSCR and siERM showing the efficiency of the knockdown. **F,** Trap force measurement of cells cultured on micropatterns and transfected or not with siRNA against ERM proteins. (N=2). **G**, Representative images of single z-planes in fixed T24 cells treated with either SCR or ERM siRNA and cultured on micropatterns. **H**, Quantification of Myo1b and Myo1c RNA by qPCR assay in ES cells treated with either SCR, siMyo1b, siMyo1c and siMyo1b+siMyo1c. Data were normalised to Actb expression. **I**, Boxes and whiskers with 10-90 percentile plot of Nanog expression of T24 cells either transfected with siMyo1b, siMyo1c or siMyo1b+siMyo1c. Only data for cells constrained on small micropatterns are shown. *P* values are calculated by Mann-Whitney U here. Graphical data represents mean ± SD unless specified otherwise. *P* values are calculated by Welch's unpaired student t-test unless specidied otherwise. Scale bars = 10μm.

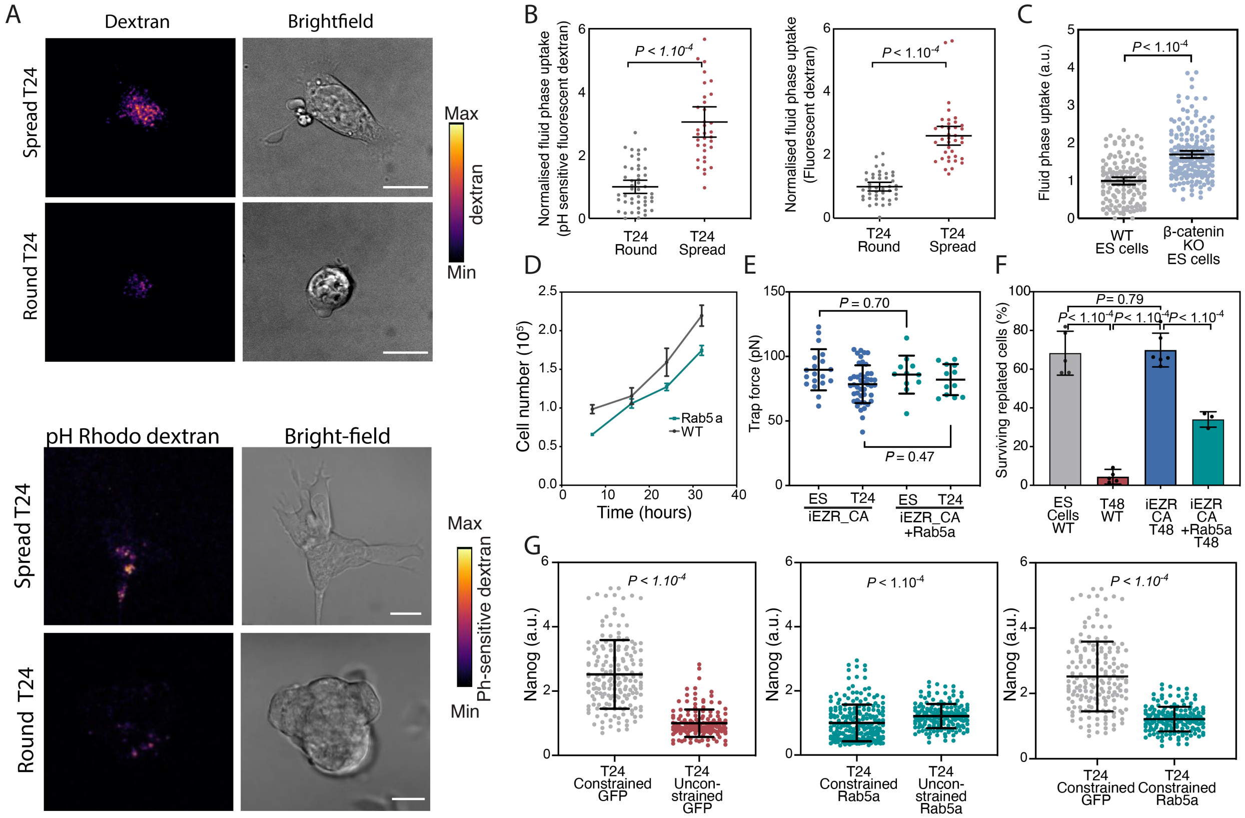

Figure S5: Endocytosis levels regulate exit from naïve pluripotency (related to Figure 5). **A**, Representative images of sum z-projections in round and spread T24 cells during a liquid phase uptake assay using fluorescent dextran or pH-sensitive fluorescent dextran. **B**, Left, quantification of liquid phase uptake using fluorescent dextran or pH-sensitive fluorescent dextran in T24 cells. Data are separated based on cell shape (round or spread). (Mean ± 95% Confidence Interval, N=2). **C,** Quantification of fluid phase uptake assay of WT & β-catenin KO ES cells. Data are normalised to WT ES cells average (N=3). **D**, Proliferation curves of WT and WT+Rab5a cells (N=2). **E**, Trap force measurements of iEZR_CA cells transfected with Rab5a, iEZR_CA trap force data are from Figure 3B. (N=2). **F**, Quantification of the percentage of surviving replated cells in a clonogenicity assay using ES and T48 cells, WT or iEZR_CA, transfected or not with Rab5a. Data for iEZR_CA T48 cells are from Figure 3E. (N=3).

G, Quantification of Nanog expression of fixed T24 cells transfected with GFP as control or Rab5a, cultured on micropatterns. This shows that Rab5a transfection can also rescue exit defects in constrained cells cultured on micropatterns. Data are normalised to cells cultured on 100 μm diameter patterns (Unconstrained) (*P* values in this panel are calculated by Mann-Whitney U test; N=3). Graphical data represents mean ± SD unless specified otherwise. *P* values are calculated by Welch's unpaired student t-test (unless specified otherwise) and are indicated in the figure. Scale bars = 10μm.

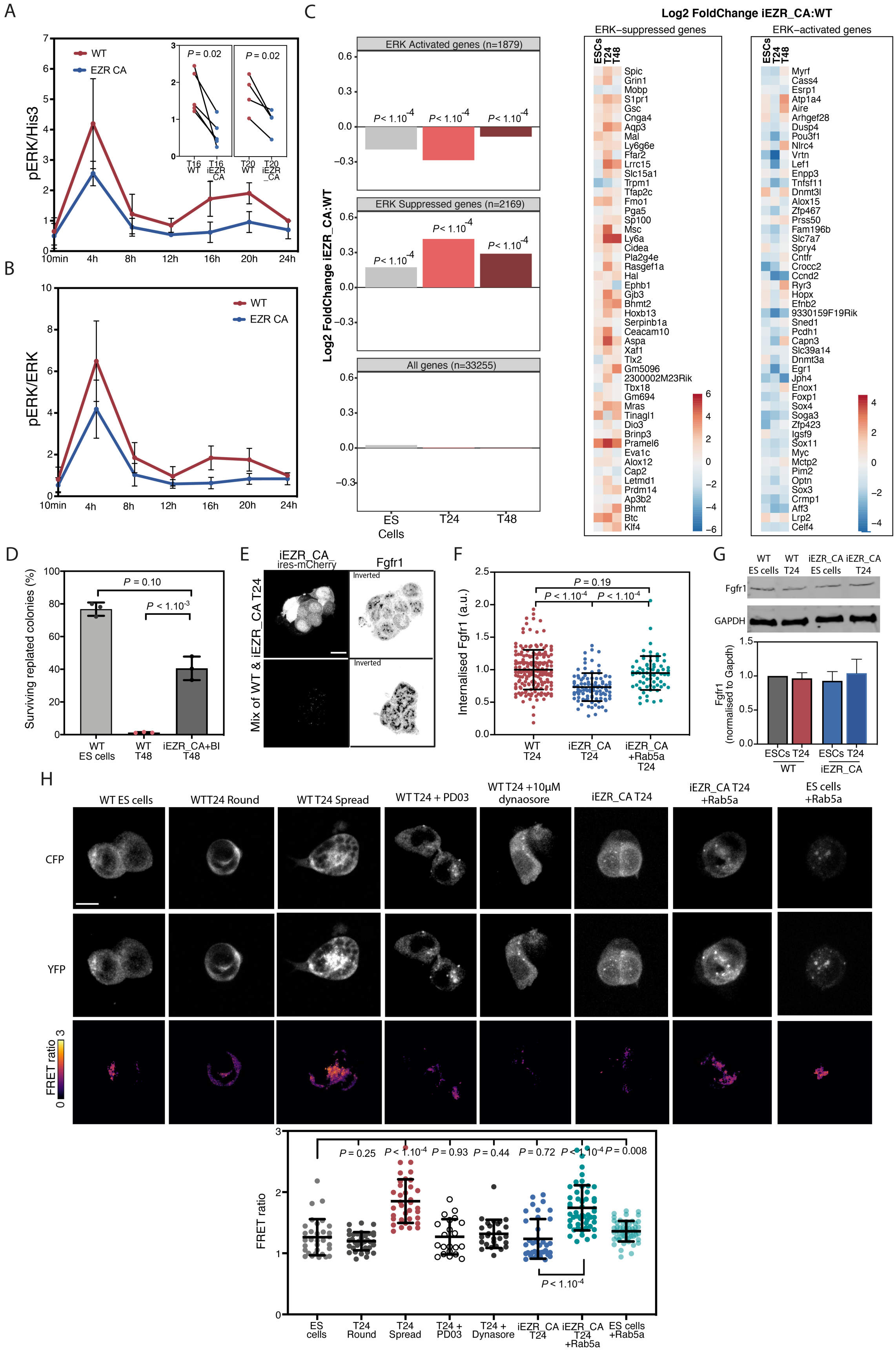
A
pERK/His3
WT
EZR CA
P = 0.02
P = 0.02
T16 WT
T16 iEZR_CA
T20 WT
T20 iEZR_CA
10min
4h
8h
12h
16h
20h
24h
B
pERK/ERK
WT
EZR CA
C
Log2 FoldChange iEZR_CA:WT
ERK Activated genes (n=1879)
ERK Suppressed genes (n=2169)
All genes (n=33255)
P< 1.10^-4
ES Cells
T24
T48
Log2 FoldChange iEZR_CA:WT
ERK−suppressed genes
ERK−activated genes
ESCs
T24
T48
Spic
Grin1
Mobp
S1pr1
Gsc
Cnga4
Aqp3
Mal
Ly6g6e
Ffar2
Lrrc15
Slc15a1
Trpm1
Tfap2c
Fmo1
Pga5
Sp100
Msc
Ly6a
Cidea
Pla2g4e
Rasgef1a
Hal
Ephb1
Gjb3
Bhmt2
Hoxb13
Serpinb1a
Ceacam10
Aspa
Xaf1
Tlx2
Gm5096
2300002M23Rik
Tbx18
Gm694
Mras
Tinagl1
Dio3
Brinp3
Pramel6
Eva1c
Alox12
Cap2
Letmd1
Prdm14
Ap3b2
Bhmt
Btc
Klf4
Myrf
Cass4
Esrp1
Atp1a4
Aire
Arhgef28
Dusp4
Pou3f1
Nlrc4
Vrtn
Lef1
Enpp3
Tnfsf11
Dnmt3l
Alox15
Zfp467
Prss50
Fam196b
Slc7a7
Spry4
Cntfr
Crocc2
Ccnd2
Ryr3
Hopx
Efnb2
9330159F19Rik
Sned1
Pcdh1
Capn3
Slc39a14
Dnmt3a
Egr1
Jph4
Enox1
Foxp1
Sox4
Soga3
Zfp423
Igsf9
Sox11
Myc
Mctp2
Pim2
Optn
Sox3
Crmp1
Aff3
Lrp2
Celf4
D
Surviving replated colonies (%)
P = 0.10
P < 1.10^-3
WT ES cells
WT T48
iEZR_CA+BI T48
E
iEZR_CA_ ires-mCherry
Fgfr1
Inverted
Mix of WT & iEZR_CA T24
F
Internalised Fgfr1 (a.u.)
P = 0.19
P < 1.10^-4
WT T24
iEZR_CA T24
iEZR_CA +Rab5a T24
G
WT ES cells
WT T24
iEZR_CA ES cells
iEZR_CA T24
Fgfr1
GAPDH
Fgfr1 (normalised to Gapdh)
ESCs T24
WT
iEZR_CA
H
WT ES cells
WTT24 Round
WT T24 Spread
WT T24 + PD03
WT T24 +10µM dynaosore
iEZR_CA T24
iEZR_CA T24 +Rab5a
ES cells +Rab5a
CFP
YFP
FRET ratio
P = 0.25
P < 1.10^-4
P = 0.93
P = 0.44
P = 0.72
P = 0.008
ES cells
T24 Round
T24 Spread
T24 + PD03
T24 + Dynasore
iEZR_CA T24
iEZR_CA T24 +Rab5a
ES cells +Rab5a

Figure S6: ERK signalling and FGFR1 expression in exit from naïve pluripotency (related to Figure 6). **A**, Quantification of phospho-ERK normalised to Histone 3 levels across several fluorescent Western blots (including Figure 6A) done on WT and iEZR_CA cells at different timepoints during exit from naïve pluripotency. The first peak of ERK activation is likely a direct effect of the removal of the inhibitors. The timing of the second peak approximately coincides with the timing of the decrease in membrane tension and cell spreading (N=5). Inset, data for T16 and T20 cells where values for WT and iEZR_CA in individual experiments are connected by a line (N=5). **B**, Similar as panel a, for phospho-ERK normalised to total ERK levels. **C,** Left, bar plot showing the average log2 fold change over all ERK-activated (n = 1879) or ERK-suppressed (n = 2169) at each of the three time-points. P-values indicate if these averages are significantly different to averages over randomly selected genes (computed using permutation test with replacement). Right, heatmap of ERK-target genes, either suppressed or activated during exit from naïve pluripotency (see methods for details). Colormap indicates the log2 fold change between expression in iEZR_CA compared to wild-type cells. For both subsets (suppressed or activated), the top 50 genes are shown (sorted based on magnitude of Erk-regulation in wild-type cells at T24). **D,** Quantification of the percentage of surviving replated cells in a clonogenicity assay using WT T48 and iEZR_CA T48 cells treated with 3µM BI-D1870. Cells replated directly from 2i+L are used as a positive control (N=6). **E,** Representative single z-plane images of a mixed population of WT and iERC_CA (positive in the mCherry channel) T24 cells, immunostained for Fgfr1. **F**, Quantification of average intracellular Fgfr1 levels in T24 cells across different conditions (N=3). **G,** Fluorescent Western blot (inverted contrast) for Fgfr1 and GAPDH in WT and iEZR_CA ES and T24 cells, and corresponding quantification (data are normalised to GAPDH). (N=3). **H**, Top, representative images of z projections of fixed ES cells in different conditions. Bottom, associated quantification of FRET ratio for each condition. *P* values are calculated by Welch's unpaired student t-test using ES cells as reference. PD03 was used at 1 µM. Dynasore was used at 10 µM. Displayed pictures have been smoothed (Gaussian blur). *P*-values are calculated by Welch's unpaired student t-test (unless specified otherwise). Graphical data represents mean ± SD unless specified otherwise. Scale bars = 10 µm.
