# Supplementary material for: Membrane Tension Gates ERK-Mediated Regulation of Pluripotent Cell Fate
Source: Cell Stem Cell. 2021 Feb 4;28(2):273–284.e6. doi: 10.1016/j.stem.2020.10.018 (PMC7875115; doi:10.1016/j.stem.2020.10.018)
Supplement: Document S2. Article plus Supplemental Information [file mmc6.pdf]

Cell Stem Cell

Article

Membrane Tension Gates ERK-Mediated Regulation of Pluripotent Cell Fate

Graphical Abstract

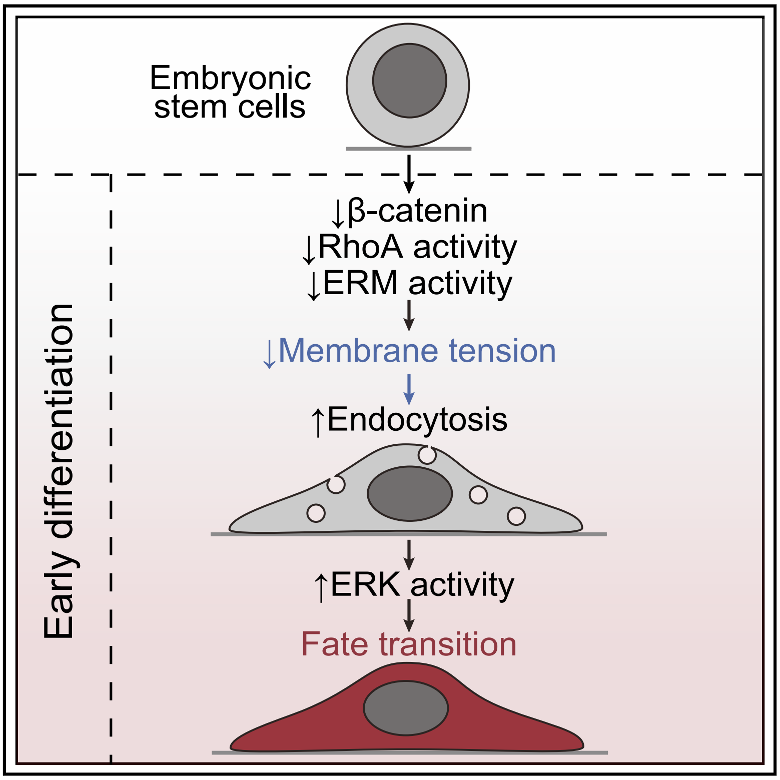

Authors

Henry De Belly, Aki Stubb, Ayaka Yanagida, Céline Labouesse, Philip H. Jones, Ewa K. Paluch, Kevin J. Chalut

Correspondence

ekp25@cam.ac.uk (E.K.P.), kc370@cam.ac.uk (K.J.C.)

In Brief

In this study, Chalut and colleagues investigate how changes in cell surface mechanics drive early differentiation in embryonic stem cells and show that preventing this change leads to developmental defects. They identify a membrane tension gated increase in endocytosis as a key mechanism regulating an ERK-regulated cell fate transition.

Highlights

- Membrane tension decreases in early differentiation of embryonic stem cells
- Membrane tension drop leads to increase in endocytic ERK signaling and fate transition
- Membrane tension drop initiated by regulatory axis, including β-catenin, RhoA, and ERM
- Preventing membrane tension drop results in developmental defects

De Belly et al., 2021, Cell Stem Cell *28*, 273–284
February 4, 2021 © 2020 The Author(s). Published by Elsevier Inc.
https://doi.org/10.1016/j.stem.2020.10.018

CellPress

Cell Stem Cell

CellPress
OPEN ACCESS

Article

Membrane Tension Gates ERK-Mediated Regulation of Pluripotent Cell Fate

Henry De Belly,[1,2,3] Aki Stubb,[3] Ayaka Yanagida,[2,4] Céline Labouesse,[2] Philip H. Jones,[5] Ewa K. Paluch,[1,3,*] and Kevin J. Chalut[2,6,*]
[1]MRC Laboratory for Molecular Cell Biology, University College London, Gower Street, London WC1E 6BT, UK
[2]Wellcome/MRC Cambridge Stem Cell Research Institute, Puddicombe Way, University of Cambridge, Cambridge CB2 0AW, UK
[3]Department of Physiology, Development, and Neuroscience, Downing Street, University of Cambridge, Cambridge CB2 3DY, UK
[4]Living Systems Institute, University of Exeter, Exeter EX4 4QD, UK
[5]Department of Physics & Astronomy, University College London, Gower Street, London WC1E 6BT, UK
[6]Lead Contact
*Correspondence: ekp25@cam.ac.uk (E.K.P.), kc370@cam.ac.uk (K.J.C.)
https://doi.org/10.1016/j.stem.2020.10.018

SUMMARY

Cell fate transitions are frequently accompanied by changes in cell shape and mechanics. However, how cellular mechanics affects the instructive signaling pathways controlling cell fate is poorly understood. To probe the interplay between shape, mechanics, and fate, we use mouse embryonic stem cells (ESCs), which change shape as they undergo early differentiation. We find that shape change is regulated by a β-catenin-mediated decrease in RhoA activity and subsequent decrease in the plasma membrane tension. Strikingly, preventing a decrease in membrane tension results in early differentiation defects in ESCs and gastruloids. Decreased membrane tension facilitates the endocytosis of FGF signaling components, which activate ERK signaling and direct the exit from the ESC state. Increasing Rab5a-facilitated endocytosis rescues defective early differentiation. Thus, we show that a mechanically triggered increase in endocytosis regulates early differentiation. Our findings are of fundamental importance for understanding how cell mechanics regulates biochemical signaling and therefore cell fate.

INTRODUCTION

The integration of mechanics with cell signaling has a crucial impact on cell function. A number of studies have highlighted the importance of sensing the mechanical properties of the extracellular environment in developmental morphogenesis (Heisenberg and Bellaïche, 2013; Koser et al., 2016), immunity (Solis et al., 2019), tumorigenesis (Halder et al., 2012), and wound healing (Razzell et al., 2014). The influence of extracellular mechanical cues is also emerging as a key player in tissue homeostasis (Gudipaty et al., 2017; Pepe-Mooney et al., 2019; Yui et al., 2018) and stem cell function and fate choice (Engler et al., 2006; Przybyla et al., 2016; Segel et al., 2019; Totaro et al., 2017). Most of these studies have focused on how the mechanics of the environment, in particular substrate stiffness, affects cell function.

At the same time, several studies suggest that changes in cell shape correlate with changes in signaling and fate. For example, it was shown that serum response factor signaling is regulated by spreading in epidermal stem cells (Connelly et al., 2010), and Stat3 signaling is linked to spreading in embryonic stem cells (Murray et al., 2013). It was also shown that the extent of spreading may affect fate choice in mesenchymal stromal cells (McBeath et al., 2004). Importantly, cell shape is controlled by forces exerted by the cytoskeleton on the cell surface; thus, it is a direct readout of intrinsic cell mechanics (subsequently cell mechanics, reviewed in Clark et al., 2014 and Heisenberg and Bellaïche, 2013). Therefore, cell mechanics and substrate stiffness could both affect signaling. However, cell and substrate mechanics are often coupled (Tee et al., 2011). As a result, studying the effects of cell mechanics on cell function is challenging, and the mechanisms by which the intrinsic mechanical properties of a cell could affect signaling remain elusive.

An excellent model system to investigate mechanical signaling in the context of a well-defined fate transition is embryonic stem cells (ESCs). Mouse ESCs possess the ability to produce all tissues, a property called pluripotency (Avior et al., 2016; Chen et al., 2016). These cells can be maintained indefinitely in a "naive" state (Evans and Kaufman, 1981; Ying et al., 2008) and ushered through distinct phases of pluripotency (Kalkan et al., 2017), including the highly characterized first stage of differentiation, exit from naive pluripotency (Kalkan et al., 2017; Mulas et al., 2019). The signaling requirements of exit from naive pluripotency are well understood; the process has been shown to be primarily driven by fibroblast growth factor/extracellular signal-regulated kinase (FGF/ERK) signaling (Kunath et al., 2007; Nett et al., 2018). Interestingly, it was recently shown that ESCs are not sensitive to external mechanical cues (Verstreken et al.,

This is an open access article under the CC BY license (http://creativecommons.org/licenses/by/4.0/).

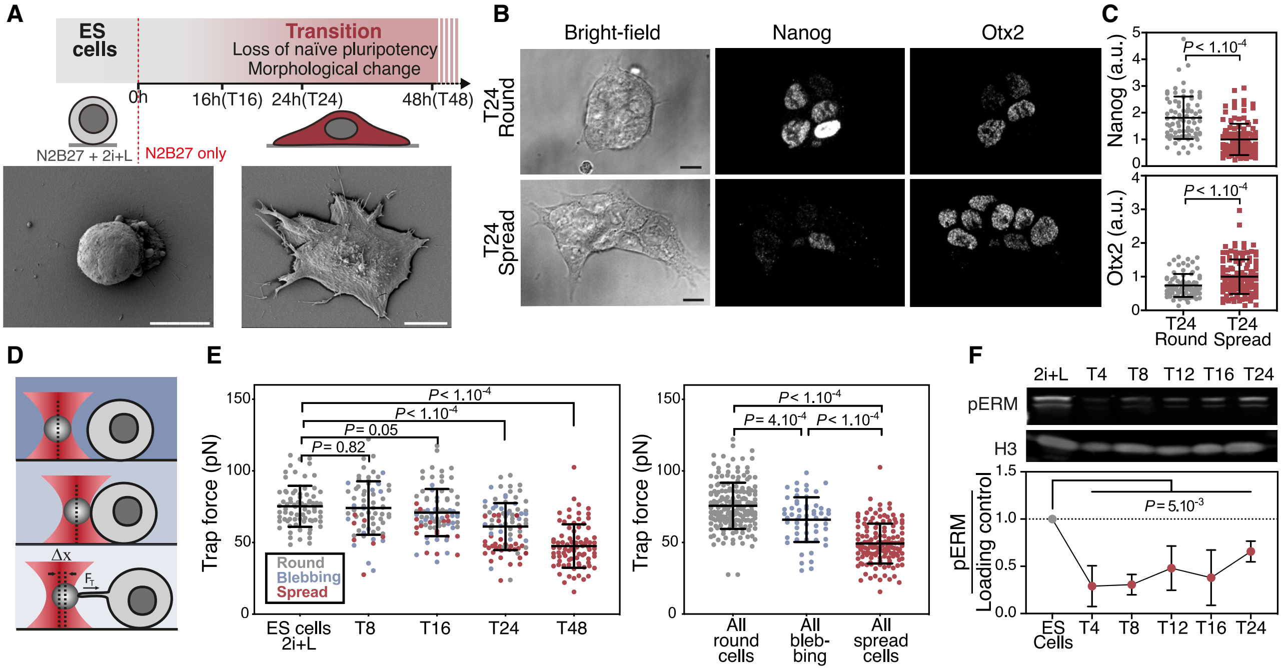

Figure 1. Membrane Tension Is Reduced in ESCs during Early Differentiation

(A) Top: schematic of experimental setup to investigate exit from naive pluripotency in ESCs; bottom: SEM images of ES and T48 cells.

(B) Representative single z planes of T24 cells immunostained for Nanog and Otx2.

(C) Quantification of Nanog and Otx2 levels (from images as in B) in round (gray dots) and spread (red dots) T24 cells (N = 3).

(D) Schematic of membrane tension measurement using optical tweezers.

(E) Left: trap force (direct readout of membrane tension) during exit from naive pluripotency of ES, T8, T16, T24, and T48 cells (means ± SDs; 5 independent experiments). The data are color coded based on cell shape (gray, round; orange, blebbing; red, spread). Right, same data as left panel but with all of the data grouped by shape (e.g., all of the blebbing cells correspond to all of the blebbing cells observed in T8, T16, and T24).

(F) Representative Western blot for pERM and His3 in ESCs and at various time points during exit from naive pluripotency and corresponding quantification (N = 4). p value was calculated using a 2-way analysis of variance (ANOVA). For all of the panels, graphical data represent means ± SDs. Unless otherwise indicated, for all panels, p values were established by Welch's unpaired Student's t test. Scale bars represent 10 μm.

2019), yet ESCs display a striking shape change, from round to spread, as they exit naive pluripotency (Chalut and Paluch, 2016), indicative of changes in cell mechanics. Thus, mouse ESCs constitute a particularly appropriate model system to dissect the role of cell mechanics in signaling in a well-defined fate transition.

Here, we show that the shape change observed in early ESC differentiation is driven by a reduction in effective membrane tension. We demonstrate that this change in membrane tension is regulated by the key pluripotency factor β-catenin (Wray et al., 2011). We further show that the decrease in membrane tension accompanying early differentiation leads to the increased endocytosis of FGF signaling components. This results in the increased activity of ERK, which is necessary for exit from naive pluripotency. Our study unveils a key mechanism whereby changes in cellular mechanics regulate the signaling that drives fate transitions.

RESULTS

Early Differentiation of ESCs Correlates with Membrane Tension Reduction and Spreading

To study the interplay between cell shape and cell fate change, we asked whether cell shape correlates with fate during the early stages of exit from naive pluripotency. ESCs were cultured on gelatin-coated plates and maintained in N2B27 medium supplemented with the mitogen-activated protein kinase kinase (MEK)/ERK inhibitor PD0325901, the glycogen synthase kinase 3 (GSK3) inhibitor/β-catenin agonist CHIRON (CHIR), and leukemia inhibitory factor (the culture medium known as 2i+L) (Mulas et al., 2019). Exit from naive pluripotency was initiated by withdrawing the inhibitors and culturing cells in N2B27 medium alone. After 24 h in N2B27 (T24), cells displayed a mixture of shapes, with most cells already flat and spread, and some still round and in colonies (Figures 1A and S1A). This heterogeneity is consistent with previously described asynchrony in exit from naive pluripotency (Kalkan et al., 2017). To identify a potential correlation with cell fate, we fixed the cells and, in cells of different shapes, quantified the expression of Nanog, a marker of naive pluripotency, and Otx2, a transcription factor upregulated in cells that have exited naive pluripotency (Kalkan et al., 2017). We found that Nanog levels were significantly higher and Otx2 significantly lower in round cells as compared to spread cells (Figures 1B and 1C), thus establishing a correlation between cell shape and exit from naive pluripotency.

We next characterized the dynamics of cell spreading during exit from naive pluripotency. To do this, we captured time-lapse images of ESCs stably expressing LifeAct-GFP to visualize

filamentous actin (Video S1). We observed that, before spreading, the cells displayed a phase of intense blebbing (Figures S1B–1D). Notably, blebbing is often a sign of low membrane-to-cortex attachment (Charras and Paluch, 2008), a key mechanical parameter controlling the apparent tension of the plasma membrane. Apparent membrane tension (subsequently referred to as "membrane tension") depends on the in-plane tension of the lipid bilayer and on membrane-to-cortex attachment, and is a measure of the resistance of the plasma membrane to deformations (Pontes et al., 2017a). As such, it is a major regulator of cell shape (Diz-Muñoz et al., 2013). Furthermore, in our scanning electron microscopy (SEM) images T48 cells displayed more surface membrane folds and reservoirs compared to naive cells (Figures 1A and S1A), suggesting differences in membrane tension (Pontes et al., 2017a).

We next asked whether exit from naive pluripotency is associated with a change in membrane tension. To address this, we used a tether pulling assay using optical tweezers (Figures 1D, 1E, and S1E; Video S2), in which the force exerted on the bead by the membrane tether (the "trap force") is a direct readout of membrane tension (Diz-Muñoz et al., 2013). We found that ESCs significantly decrease their membrane tension as they exit naive pluripotency (Figure 1E). Moreover, at T24, when cell populations display mixed morphologies, round cells had a significantly higher membrane tension than blebbing and spread cells. This is consistent with previous studies showing that cell spreading is facilitated by decreased membrane tension (Pontes et al., 2017b; Raucher and Sheetz, 2000). Therefore, we conclude that cell spreading during exit from naive pluripotency occurs concomitantly with a decrease in plasma membrane tension.

Membrane tension has been shown to be regulated largely by linkers between the plasma membrane and the underlying actomyosin cortex, such as ezrin-radixin-moesin (ERM) and myosin I proteins (Nambiar et al., 2009; Pontes et al., 2017a). To narrow the list of potential regulators of membrane tension, we checked the expression of genes encoding for linker proteins in ESCs and in their *in vivo* counterpart, the pre-implantation epiblast, using a published dataset (Boroviak et al., 2015). We found that ERM proteins, and in particular Ezrin, were up to 10 times more expressed than myosin I proteins. We thus focused on ERM proteins, which are activated by phosphorylation (Gautreau et al., 2000). At the population level, we found that the level of phosphorylated ERM (pERM) was sharply decreased after 2i+L removal (Figures 1F and S1F). We confirmed these results using immunostaining of T16 cells, which showed that spread cells have lower levels of pERM than round cells (Figures S1G–S1J).

The Decrease in Membrane Tension during Early Differentiation Is Induced by a β-Catenin-Mediated Decrease in ERM Phosphorylation

We then investigated which pluripotency-regulating signaling pathway is primarily responsible for the decrease in pERM upon removal of ESC media. We reduced the medium to the minimal signaling environment necessary to maintain naive pluripotency (2i) (Ying et al., 2008). We then separately removed PD0325901 (PD03) and CHIR from 2i to study the effects of MEK/ERK activation and GSK3b activation, respectively. We found that, while PD03 removal did not lead to a pERM and decrease, CHIR removal resulted in a rapid and significant decrease in pERM and decrease in membrane tension (Figures S2A–S2C), pointing to a role for GSK3b signaling in regulating pERM levels. Given that increased GSK3b activation leads to β-catenin degradation (Liu et al., 2002), and that β-catenin is partly localized at the plasma membrane, we asked whether β-catenin depletion would lead to a decrease in pERM and subsequent decrease in membrane tension. To address this question, we measured pERM levels and membrane tension in β-catenin knockout (KO) ESCs (Wray et al., 2011) and found that both were significantly lower compared to wild-type (WT) ESCs (Figures 2A and 2B). These results suggest that GSK3b-driven β-catenin degradation mediates a decrease in pERM, which in turn controls membrane tension and cell shape during exit from naive pluripotency.

β-Catenin Regulates ERM Phosphorylation by Modulating RhoA Activity

We next investigated the link between β-catenin and ERM phosphorylation. Given that there is a known link between RhoA activity and the cadherin-catenin complexes (Arnold et al., 2017) and that active RhoA is one of the key activators of ERMs (Matsui et al., 1998), we speculated that RhoA may connect β-catenin to ERM. To test this hypothesis, we measured RhoA activity during exit from naive pluripotency using an active fluorescence resonance energy transfer (FRET) reporter and an active RhoA pull-down assay and found that ESCs displayed a significant decrease in RhoA activity during exit from naive pluripotency (Figures 2C–2F and S2D). Furthermore, our results show that the levels of active RhoA are significantly lower in β-catenin KO cells compared to WT ESCs (Figures 2E and 2F).

We then used a doxycycline-inducible ESC line expressing a phosphomimetic, constitutively active form of RhoA to probe whether higher RhoA activity does indeed lead to higher ERM phosphorylation, and thus higher membrane tension in ESCs. After doxycycline induction, we found that inducible, constitutively active RhoA (iRhoA_CA) cells showed high levels of ERM phosphorylation and subsequently maintained high membrane tension during exit from naive pluripotency (Figures 2G and 2H). Our findings indicate that β-catenin degradation by GSK3 leads to a decrease in RhoA activity, which drives a subsequent decrease in ERM phosphorylation and membrane tension, suggesting that RhoA activity represents a key link between β-catenin and ERM regulation.

Maintaining High Membrane Tension Impairs Early Differentiation

We then asked whether the membrane tension decrease plays a role in controlling exit from naive pluripotency. We used both the iRhoA_CA cells and a doxycycline-inducible ESC line expressing a phosphomimetic, constitutively active form of ezrin (iEZR_CA). After induction, iEZR_CA cells showed high EZR_CA expression and no growth defects (Figures S3A and S3B). After withdrawal of the naive factors (2i+L), iEZR_CA and iRhoA_CA cells did not spread (Figures 3A and S3C), even at T48, and they maintained high membrane tension similar to ESCs (Figures 2H and 3B). We also found that, compared to controls, iEZR_CA and

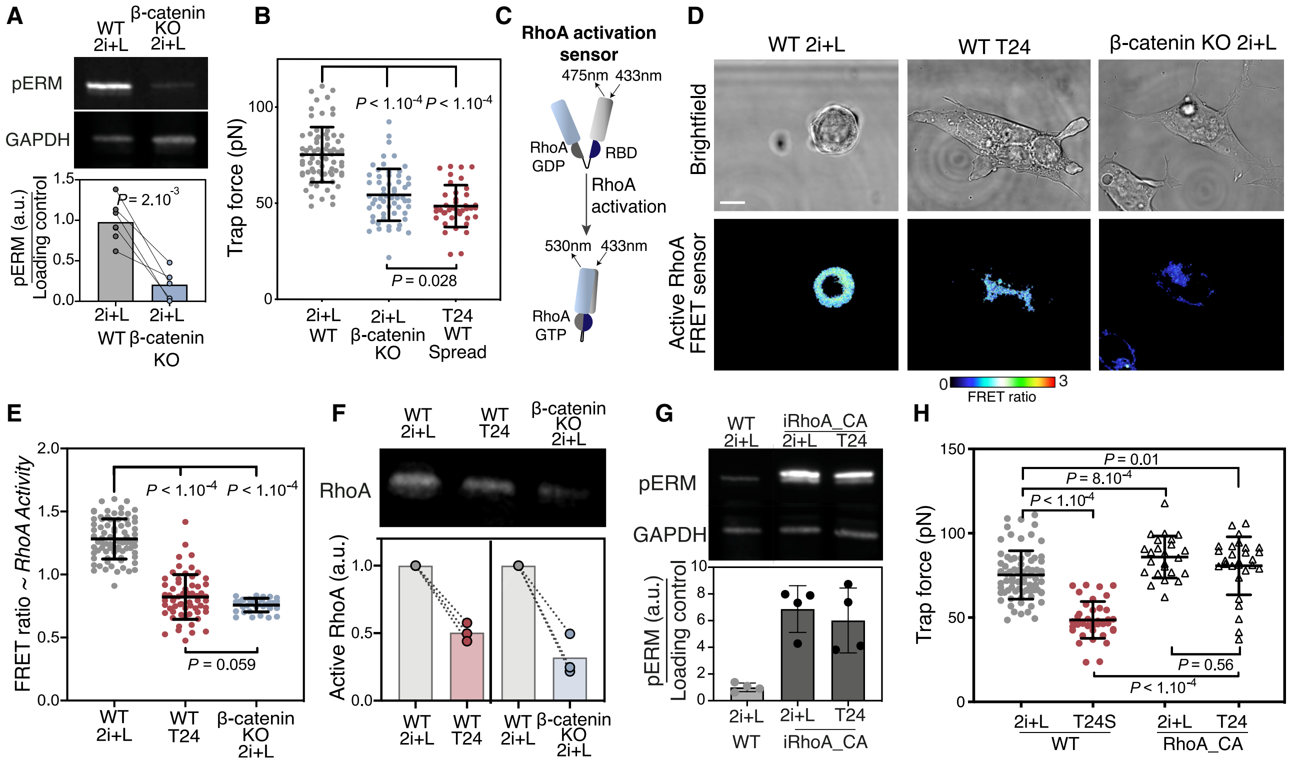

Figure 2. The Decrease in Membrane Tension during Early Differentiation Is Induced by a β-Catenin and RhoA-Mediated Decrease in ERM Phosphorylation

(A) Fluorescent Western blot and associated quantification for pERM and glyceraldehyde 3-phosphate dehydrogenase (GAPDH) of WT and β-catenin knockout (KO) cells cultured in 2i+L (N = 6).

(B) Trap force measurements of β-catenin KO ESCs and WT ESCs and T24 spread (S) cells (N = 3).

(C) Schematic of the FRET sensor for RhoA activity. RBD, Rho binding domain.

(D) Representative images of the bright-field and FRET ratio of WT ESCs, T24 cells, and β-catenin KO ESCs expressing the RhoA activation FRET sensor.

(E) Quantification of the average FRET ratio (~RhoA activity) per cell (N = 3).

(F) Active RhoA pull-down assay. Top, representative fluorescent Western blot for RhoA in WT ESCs, T24, and β-catenin KO cells after active RhoA pull-down. Bottom, quantification of active RhoA pulled down. (N = 3).

(G) Top, representative fluorescent Western blot for pERM and GAPDH in WT ESCs, iRhoA_CA ESCs, and T24 cells. Note that WT 2i+L is the same as in (A) (A and G are on the same gel). Bottom, corresponding quantification (N = 4).

(H) Trap force measurement of WT ESCs, T24S, iRhoA_CA ESCs, and T24 cells (N = 3). WT data in (B) and (H) are from Figure 1E. The graphical data represent means ± SDs. p values established by Welch's unpaired Student's t test and indicated in the figure. Scale bars represent 10 μm.

iRhoA_CA cells at T24 maintained significantly higher levels of Nanog and failed to efficiently upregulate Otx2 (Figures 3C, 3D, S3C, and S3D). A clonogenicity assay indicated that unlike control T48 WT cells, iEZR_CA and iRhoA_CA cells placed back in 2i+L after 48 h in N2B27 were able to survive and form naive colonies with the same efficiency as naive ESCs (Figures 3E and S3E; see Method Details). To confirm the early differentiation phenotype, we performed RNA sequencing (RNA-seq) comparing iEZR_CA and WT ESC, T24, and T48 cells. We found that iEZR_CA cells showed a significant impairment both in loss of naive pluripotency markers and subsequent expression of early implantation markers (Figures 3F, 3G, and S3I; Table S1). These results indicate that maintaining a high membrane tension in pluripotent cells significantly inhibits their ability to exit naive pluripotency.

Furthermore, treating cells with methyl-β-cyclodextrin, a cholesterol-sequestering compound that increases membrane tension (Biswas et al., 2018; Figures S3J and S3K), delayed exit from naive pluripotency (Figure S3L). Conversely, treating cells with NSC 668394, an ezrin inhibitor that decreases membrane tension (Rouven Brückner et al., 2015; Figures S3J and S3K), caused cells to exit naive pluripotency more efficiently than control cells (Figure S3L). Moreover, a recent study showed that using a purely mechanical linker between actin and the plasma membrane to increase effective membrane tension also blocks exit from naive pluripotency (Bergert et al., 2020, this issue of *Cell Stem Cell*).

To assess the developmental relevance of these findings, we used a gastruloid assay (adapted from Baillie-Johnson et al., 2015) in which we compared the ability of iEZR_CA to form gastruloids at different stages (Figures 3H–3K). We found that iEZR_CA gastruloids displayed clear morphological defects, failing to elongate and adopt the characteristic morphology of properly progressing gastruloids with distinct multiaxial organization. The lack of elongation suggests major developmental defects in these gastruloids (Turner et al., 2017). We also performed

CellPress
OPEN ACCESS

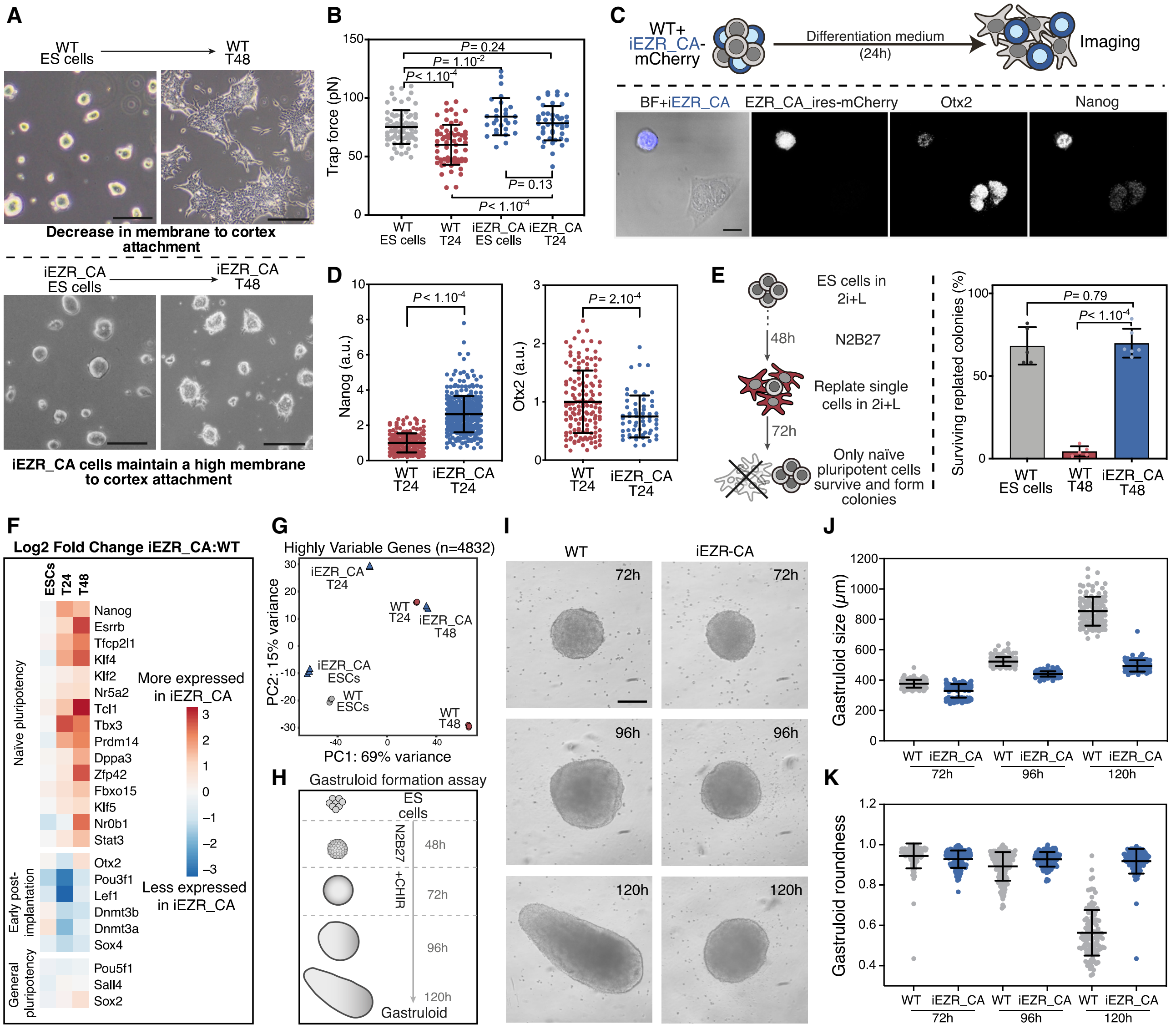

Figure 3. Maintaining High Membrane Tension Impairs Early Differentiation

(A) Representative images of WT (top) and iEZR_CA (bottom) ESCs and T48 cells. Scale bar, 50 μm.

(B) Trap force, as a readout of membrane tension, for WT and iEZR_CA ESCs and T24 cells (N = 3; data for WT cells are same as Figure 1H).

(C) Representative single z planes of a mix of WT and iEZR_CA (positive for the EZR_CA_ires_mCherry) T24 cells immunostained for Otx2 and Nanog. Scale bar represents 10 μm.

(D) Quantification of Nanog and Otx2 expression in WT and iEZR_CA T24 cells, normalized to WT mean levels (N = 3).

(E) Left: schematic of clonogenicity assay used as a functional measure of naive pluripotency. Right: quantification of the percentage of surviving replated cells in a clonogenicity assay using WT and iEZR_CA ESs. Cells replated directly from 2i+L are used as a positive control (N = 6).

(F) Heatmap of relative expression of main pluripotency genes and early post-implantation genes. The mean normalized log2 counts for each time point in iEZR_CA cells is compared to mean normalized log2 counts in WT cells. Averages were computed over 3 biological replicates.

(G) Principal-component analysis from RNA sequencing of iEZR_CA and WT ESCs in 2i+LIF, and 24 h (T24) and 48 h (T48). Each marker represents an independent biological replicate (3 replicates per condition). The principal components ("PC") were computed based on the normalized expression of highly variable genes (n = 4,832 genes) (see Method Details).

(H) Schematic presentation of the gastruloid culture protocol: 300 mouse ESCs were transferred into low-attachment wells. CHIR99021 was introduced from 48 to 72 h. Organoids were cultured for a total of 120 h and images acquired at 72, 96, and 120 h time points.

(I) Representative bright-field microscope images of gastruloids initiated from WT cells (left) and iEZR_CA cells (right).Scale bar, 200 um. (J and K) Quantification of WT and iEZR_CA gastruloid size and shape. Maximum feret diameter and roundness (see STAR Methods) measured from the brightfield images taken at 72 h, 96 h, and 120h timepoints (N = 3). Graphical data represents mean ± SD. p values established by Welch's unpaired student t test (unless specified otherwise).

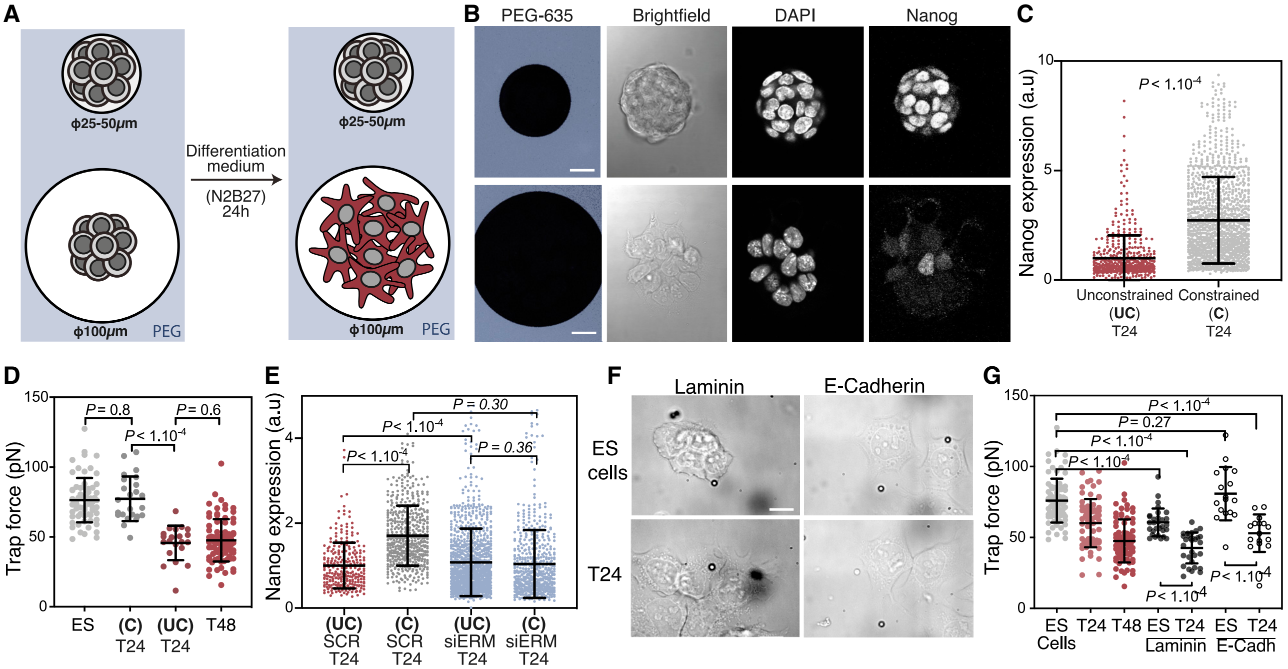

Figure 4. Membrane Tension Reduction, Not Cell Spreading, Is Responsible for Gating Early Differentiation

(A) Schematic of the micropatterning assay. Cells cannot adhere on polyethylene glycol (PEG) regions (blue) and can only adhere on the micropatterns.

(B) Representative single z plane images of fixed ES and T24 cells cultured on small (top) and large (bottom) micropatterns and immunostained for Nanog.

(C) Quantification of Nanog expression in ES and T24 cells cultured on large (unconstrained, control) and small (constrained) micropatterns (here, p values were calculated using the Mann-Whitney *U* test, N = 4).

(D) Membrane tension measurements in ESCs and T24 cells either grown on micropatterns (small, constrained; large, unconstrained) or cultured in an open gelatin-coated dish (ES and T48). The data for cells on gelatin are from Figure 2B (N = 2).

(E) Similar quantifications as in (C), but for cells transfected with triple small interfering RNA (siRNA) against ERM or with Scrambled (SCR) siRNA as control (Mann-Whitney *U* test was used to calculate the p value; N = 4).

(F) Representative images of ES and T24 cells cultured on laminin and E-cadherin.

(G) Trap force measurements in ESCs and T24 cells cultured on either gelatin, laminin, or E-cadherin. The data for cells cultured on gelatin are the same as Figure 2B (N = 3). The graphical data represent means ± SDs. Unless otherwise specified, the p values were calculated using Welch's unpaired Student's t test. Scale bars represent 10 μm.

morula injection of iEZR_CA and control cells at the 8-cell stage and maintained embryos in culture for 2 or 3 days, resulting in embryos that are the equivalent of developmental stages embryonic days (E)4.5 and E5.5, respectively. We consistently observed a higher level of the naive pluripotency marker Nanog in the iEZR_CA compared to native and control cells (Figures S4A and S4B), thus suggesting that high membrane tension also leads to a delay in differentiation in the embryo.

Our data strongly suggest that a pERM-controlled decrease in plasma membrane tension is essential for cell spreading and for exit from naive pluripotency.

Membrane Tension Reduction, Not Cell Spreading, Is Responsible for Gating Early Differentiation

To clarify the relative importance of cell shape change and membrane tension, we used micropatterning to control the extent of cell spreading with disks of different diameters (small: 25–50 μm or large: 100 μm). We seeded ESCs at equal densities onto both small and large micropatterns in N2B27 only. We found that in contrast to cells plated on large patterns or non-patterned surfaces, spatially constrained T24 cells on small patterns did not change shape and maintained a rounded morphology similar to ESCs (Figures 4A and 4B). Spatially constrained cells at T24 maintained high levels of Nanog, suggesting defects in early differentiation (Figures 4B, 4C, and S4C). However, spatially constrained cells also maintained a high membrane tension (Figure 4D). Strikingly, the exit from naive pluripotency on small micropatterns, where cells cannot spread, was rescued by knockdown of ERM (Figures 4E and S4D–S4G) or knockdown of myosin I family proteins (Figures S4H and S4I), treatments previously shown to reduce membrane tension (Diz-Muñoz et al., 2010; Rouven Brückner et al., 2015). Together, these experiments point to the existence of positive feedback loops between membrane tension decrease and cell spreading, and indicate that it is the decrease in membrane tension, rather than cell spreading, that is required for efficient exit from naive pluripotency.

To further test whether membrane tension regulates exit from naive pluripotency directly or as a result of cell spreading, we plated ESCs on either laminin or E-cadherin, where they display spread morphologies even in 2i+L (Figure 4F). Despite the fact that for cells on laminin and E-cadherin exit from pluripotency was associated with a much less pronounced spreading increase than for cells on gelatin, we still observed a similar drop in membrane tension during exit from pluripotency (Figure 4G).

CellPress
OPEN ACCESS

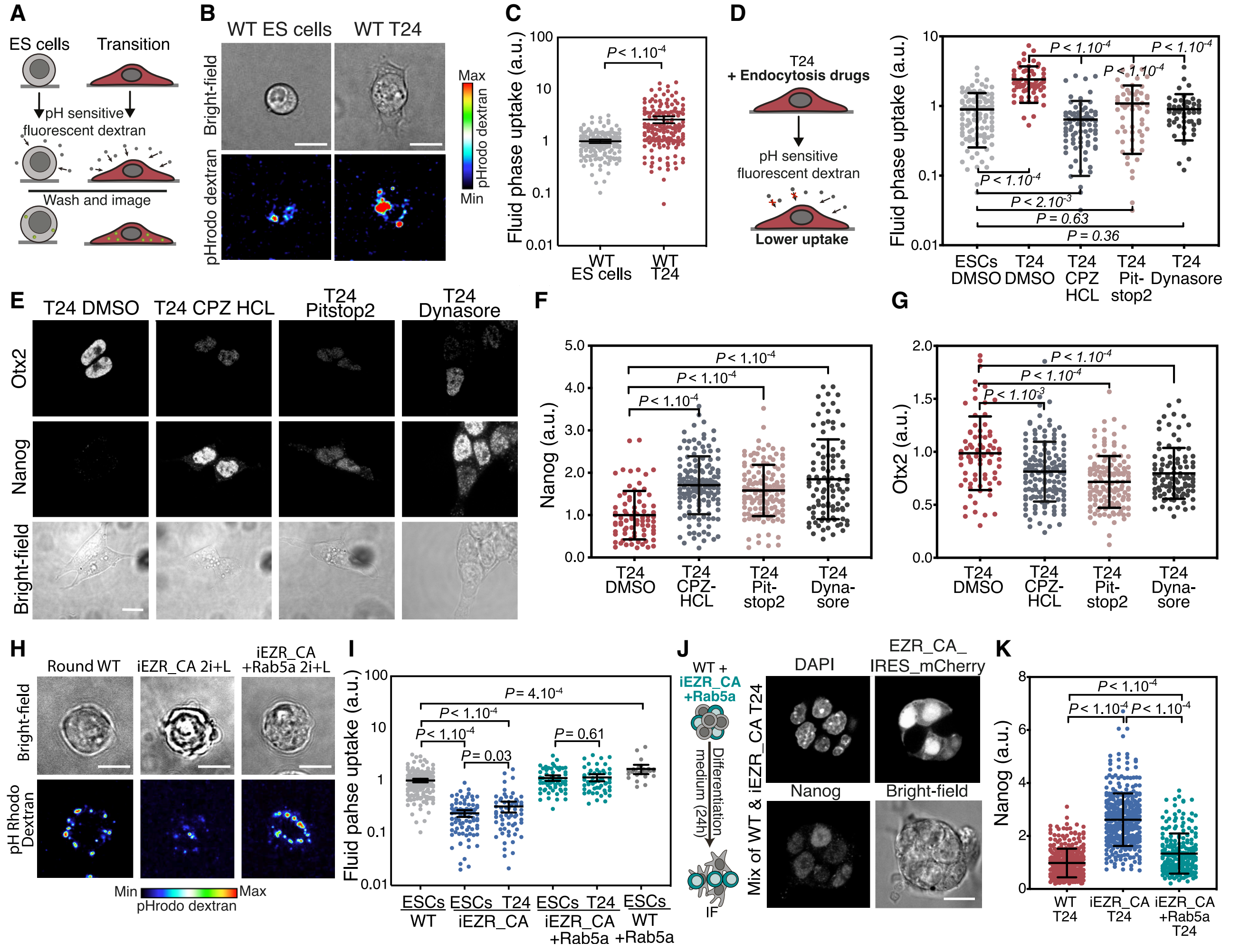

Figure 5. Endocytosis Regulates Early Differentiation

(A) Schematic of endocytosis quantification using a fluid uptake assay with pH-sensitive fluorescent dextran.

(B and C) Sum z projection images of representative ESCs and T24 cells in assay described in (A) and (B) and corresponding quantification (C; error bars are 95% confidence intervals; N = 3).

(D) Left: schematic of endocytosis quantification assay with the use of drug treatment against endocytosis. Right: quantification of fluid phase uptake in ESCs and T24 cells treated with either DMSO (control), chlorpromazine hydrochloride (10 μM), pitstop2 (25 μM), or dynasore (10 μM).

(E) Representative images of immunofluorescence against Nanog and Otx2 of a single z plane of T24 cells treated with either DMSO (control), chlorpromazine hydrochloride (10 μM), pitstop2 (25 μM), or dynasore (10 μM).

(F and G) Quantification of Nanog and Otx2 expression in cells treated with drugs to inhibit endocytosis, normalized to control (DMSO) mean levels (N = 3).

(H and I) Sum z projection images of representative WT ESCs, iEZR_CA-ESCs, and iEZR_CA-ESCs transfected with Rab5a in the assay described in (A) and associated quantifications. Error bars: 95% confidence intervals (N = 3).

(J and K) Single z plane images of representative mixed populations of WT and iEZR_CA (positive in the mCherry channel) T24 cells, transfected with Rab5a and immunostained for Nanog and corresponding quantifications (N = 3). The non-transfected iEZR_CA data are from Figure 2D. The graphical data represent means ± SDs. p values calculated using Welch's unpaired Student's t test. Scale bars represent 10 μm.

This further indicates that it is the decrease in membrane tension, and not the spreading directly, that is needed for efficient dismantling of naive pluripotency.

Membrane Tension Regulates Endocytosis Levels in ESCs

We then asked how decreased membrane tension facilitates exit from the ESC state. We speculated that the facilitation may be through endocytosis, which is a major regulator of signaling events (Pouille et al., 2009; Sorkin and von Zastrow, 2009) and has been shown to be regulated by membrane tension (Dai and Sheetz, 1995; Thottacherry et al., 2018). We assessed global endocytosis levels using a fluid phase uptake assay, in which we cultured cells in the presence of either fluorescent dextran or a pH-sensitive fluorescent dextran (Figure 5A). Using this assay, we found that T24 cells displayed a significant increase in endocytosis levels once spread (Figures 5B, 5C, S5A, and S5B). We next interfered with this increase using drugs known to inhibit endocytosis (dynasore, pitstop2, and chlorpromazine hydrochloride) (Dutta and Donaldson, 2012).

We found that WT cells treated with these drugs failed to increase their endocytosis rates during exit from naive pluripotency (Figure 5D). We next assessed whether cells with endocytosis defects could exit naive pluripotency normally and found that these cells maintained a higher level of Nanog and a lower level of Otx2 expression compared to controls (Figures 5E–5G). These observations suggest that endocytosis regulates exit from naive pluripotency.

We next examined the link between the observed differentiation-induced decrease in membrane tension and endocytosis. We showed that endocytosis was considerably reduced in cells expressing iEZR_CA (Figures 5H and 5I) and that it was increased in β-catenin KO ESCs (Figure S5C), suggesting (Dai and Sheetz, 1995; Thottacherry et al., 2018) that high membrane tension antagonizes endocytosis, which is consistent with previous findings in other systems. To test whether enhanced endocytosis could overcome the block in differentiation imposed by EZR_CA, we overexpressed Rab5a, a master regulator of early endosomes (Palamidessi et al., 2019), in iEZR_CA cells. The elevation of Rab5a levels has been shown to promote endocytosis (Mendoza et al., 2014; Palamidessi et al., 2019). We found that overexpressing Rab5a increased endocytosis levels in iEZR_CA cells compared to non-transfected controls, both in ESCs and T24 cells without leading to growth defects (Figures 5H, 5I, and S5D) and without lowering membrane tension (Figure S5E). Notably, Rab5a expression rescued the early differentiation defect phenotype in iEZR_CA cells, restoring Nanog downregulation (Figures 5J and 5K) and decreasing the number of colonies surviving in a clonogenicity assay, which is indicative of more efficient exit from naive pluripotency (Figure S5F). Furthermore, transfection with Rab5a also enabled the downregulation of Nanog protein in spatially constrained T24 cells (Figure S5G). These results show that increasing endocytosis levels via Rab5a overexpression allows ESCs to exit naive pluripotency notwithstanding a high membrane tension.

Membrane-Tension-Mediated Endocytosis Promotes ERK Activation during Early Differentiation

Finally, we asked whether a specific signaling pathway related to the regulation of naive pluripotency was affected by the changes in cell mechanics and endocytosis that we observed. The most obvious candidate was FGF/ERK, as it is key for exit from naive pluripotency (Nichols et al., 2009), and its EGF/ERK counterpart been shown to be regulated by endocytosis in cancer cells (Palamidessi et al., 2019; Sorkin and von Zastrow, 2009). We thus quantified pERK levels during exit from naive pluripotency and observed that at all time points ERK activity was highly attenuated in the iEZR_CA cells (Figures 6A, 6B, S6A, and S6B). We also confirmed a global reduction in mitogen-activated protein kinase (MAPK)/ERK signaling in iEZR_CA cells by RNA-seq analysis of known transcriptional targets (Figure S6C). These results strongly suggest that maintaining high membrane tension during exit from naive pluripotency prevents ERK activation. To further test the role of ERK activity in the early differentiation impairment of the iEZR_CA cells, we treated iEZR_CA cells with the p90 ribosomal S6 kinase (RSK) inhibitor BI-D1870, which acts as an ERK activator (Nett et al., 2018). We found that this treatment was sufficient to rescue the early differentiation defects of iEZR_CA (Figures 6C and 6D), which we further confirmed using a clonogenicity assay (Figure S6D).

The internalization of receptors by endocytosis has been shown to influence the levels of pERK activity (Palamidessi et al., 2019; Platta and Stenmark, 2011). Thus, we measured the internalization of FGF receptor 1 (FGFR1), which is upstream of pERK in ESCs (Molotkov et al., 2017), iEZR_CA, and control cells using immunofluorescence. We found that iEZR_CA cells had less internalized FGFR1 compared to controls (Figures S6E and S6F), despite expressing similar protein levels (Figure S6G). We were able to increase the levels of internalized FGFR1 in iEZR_CA by overexpressing Rab5a (Figures S6E and S6F). We then specifically probed pERK activity at endosomes using a recently described FRET sensor reporting on ERK activity at early endosomes (Figure 6E; Palamidessi et al., 2019). Using live imaging, we found that pERK at endosomes sharply increased as cells spread (Figures 6E–6G and S6H; Video S3). We confirmed this with measurements in fixed cells, finding that spread T24 cells showed a higher FRET ratio compared to round T24 cells. Notably, we observed a lower FRET ratio in T24 cells, where endocytosis rates were reduced chemically with dynasore or by increasing membrane tension via iEZR_CA expression (Figure 6H). Furthermore, increasing endocytosis independently of membrane tension by expressing Rab5a in iEZR_CA cells, resulted in a FRET ratio similar to spread control cells at T24 (Figure 6H). Thus, our results indicate that ERK activity, which helps disassemble naive pluripotency, is induced by membrane tension-regulated endocytosis in cells exiting pluripotency (Figure 6I).

DISCUSSION

There is extensive interplay between substrate mechanics, cell mechanics, and cell function (Guilak et al., 2009). Moreover, cell shape, which is a readout of cell mechanics, is correlated with cell fate (Bellas and Chen, 2014). Because cell shape, cell mechanics, and cell fate are deeply intertwined, it is challenging to disentangle them to understand specifically how cell mechanics mediates signaling. To probe the role of cell mechanics in cell fate transitions, we focused on exit from naive pluripotency in ESCs. Interestingly, we found that it is the change in cell surface mechanics, not the cell shape, that regulates early differentiation. We found that membrane tension decreases as ESCs exit naive pluripotency, and prohibiting ESCs from spreading inhibited both membrane tension reduction and early differentiation. Decreasing membrane tension in constrained cells was sufficient to rescue the differentiation defect. These results suggest an important feedback between cell shape and cell surface mechanics during the early differentiation of ESCs but indicate that here it is cell surface mechanics that constitutes the primary mediator of cell fate transitions.

Our results also suggest a previously unknown synergy between essential pluripotency regulators β-catenin and FGF/ERK. This synergy demonstrates clear feedback between cell signaling and surface mechanics. We show that β-catenin regulates membrane tension, which in turn gates downstream signaling activity by regulating endocytosis. We speculate that β-catenin regulates membrane tension by modulating RhoA activity via the cadherin-catenin complex, which has been shown to regulate various Rho GTPases (Arnold et al., 2017). In this model, upon the initiation of

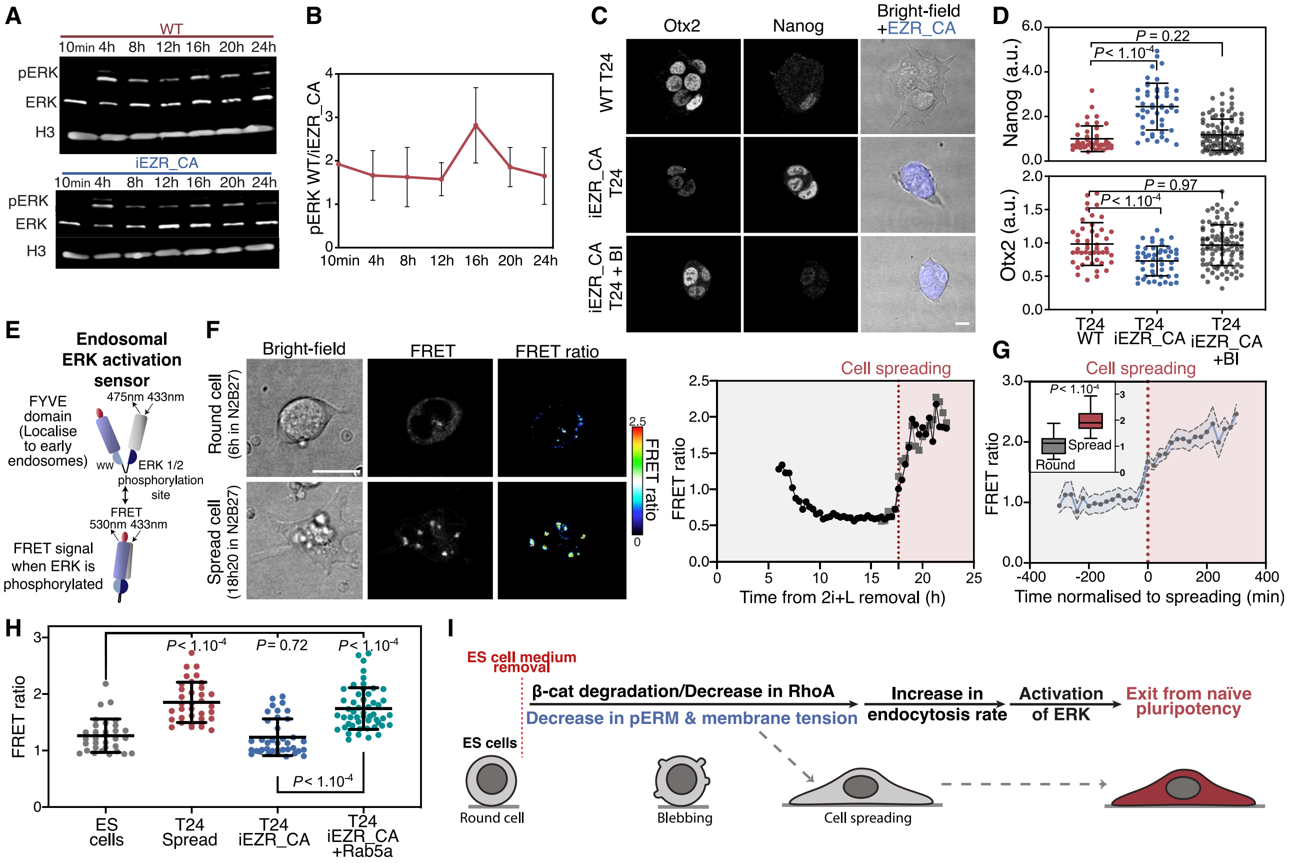

Figure 6. Membrane-Tension-Mediated Endocytosis Promotes ERK Activation during Early Differentiation

(A) Fluorescent Western blot for ERK, pERK, and histone3 in ESCs at different time points during exit from naive pluripotency.

(B) Time course of the ratio of pERK levels (normalized to corresponding histone 3 levels) in WT and iEZR_CA cells during exit from naive pluripotency (N = 6).

(C) Representative single z plane image of WT, iEZR_CA, and iEZR_CA T24 treated with 3 μM BI-D1870, an RSK-inhibitor (resulting in ERK activation) immunostained for Nanog and Otx2.

(D) Quantification of Nanog and Otx2 expression in WT, iEZR_CA, iEZR_CA+BI-D1870 T24 cells, normalized to WT mean levels (N = 3).

(E) Schematic of the FRET ERK sensor used to measure ERK activation at early endosomes (Palamidessi et al., 2019).

(F) Left: time-lapse of a representative cell expressing the FRET ERK sensor and exiting pluripotency at 6 h (top, the cell is still round) and 18 h 20 min (bottom, the cell is spread). Right: time course of the FRET ratio for the cell is shown at left.

(G) Quantification of the FRET ratio in ESCs exiting naive pluripotency; time is normalized to the time of spreading (means ± SEMs, n = 10, N = 3). Inset: FRET ratio for round and spread cells in the same dataset.

(H) Mean FRET ratio for fixed ESCs and T24 cells in WT and iEZR_CA transfected or not with Rab5a (N = 3).

(I) Schematic of the proposed mechanism for how membrane tension regulates signaling during exit from naive pluripotency. The graphical data represent means ± SDs. p values calculated using Welch's unpaired Student's t test (unless specified otherwise) and indicated in the figure. Scale bars represent 10 μm.

early differentiation, degradation of β-catenin by GSK3 leads to a decrease in RhoA activity and subsequent decrease in ERM phosphorylation/membrane tension. Ultimately, our results suggest a role for β-catenin in maintaining naive pluripotency by preserving high membrane tension, which would thus suppress endocytic FGF/ERK signaling in naive cells.

Previous studies have pointed to correlations between mechanical changes and endocytosis, with links to developmental processes (Pouille et al., 2009). Furthermore, osmotic changes, which among other activities affect membrane tension, have been linked with the endocytosis of receptors, leading to the inhibition of signaling pathways during transdifferentiation of muscle progenitors (Rauch et al., 2002). In contrast, our study demonstrates that in ESCs, FGF signaling is activated by increased endocytosis as a result of changes in cell surface mechanics. By unveiling a specific pathway activated as a result of a change in membrane tension, our study draws a direct link between cell mechanics, endocytic signaling, and fate choice.

Our findings point to plasma membrane tension as a central mediator of cell signaling. As the plasma membrane defines the boundary between the inside and outside of the cells, it is an ideal cellular element to integrate environmental cues and mechanical changes into changes in cell signaling. Furthermore, we speculate that observed effects of substrate mechanics on stem cell fate regulation in other systems (Engler et al., 2006; Przybyla et al., 2016) may be due in part to the resulting changes in membrane tension and downstream endocytosis rate. Going forward, it will be important to assess whether and how the stiffness of the microenvironment can affect membrane tension. Given that many morphogenetic processes in development are accompanied by

significant shape changes (Paluch and Heisenberg, 2009), it will also be interesting to further explore this connection *in vivo*. Our work unveils a mechanism directly connecting a specific change in cell mechanics to the activation of a key biochemical signaling pathway. The interplay between mechanics, membrane tension, and endocytic signaling is likely to be an important regulator of cell fate in various developmental and pathological contexts.

Limitations of Study

A limitation of our study is the lack of quantitative three-dimensional (3D) cell shape measurements. In our study, we classified cells by shape using two categories: round and spread. Quantitative cell shape measurement may allow us to identify an intermediary state between round and spread. Also, as described in this study and others, differentiation is an asynchronous process. As such, the bulk assays (e.g., Western blots, RNA-seq) used in our investigation may lead to averaging out transient mixed populations.

STAR★METHODS

Detailed methods are provided in the online version of this paper and include the following:

- KEY RESOURCES TABLE
- RESOURCE AVAILABILITY
  - Lead Contact
  - Materials Availability
  - Data and Code Availability
- EXPERIMENTAL MODEL AND SUBJECT DETAILS
  - Cell lines
  - Mice and mice embryos
- METHOD DETAILS
  - Cell culture, transfections and exit from naive pluripotency
  - Tether pulling and trap force measurements
  - RNA extraction and qPCR
  - Western blots
  - Immunofluorescence
  - RNA sequencing, data processing, differential expression analysis and gene ontology analysis
  - Micropatterning
  - Generation of chimeras
  - Isolation of ICMs from embryos, and *in vitro* culture
  - RhoA pulldown assay
  - Immunofluorescence staining
  - Gastruloid culture
  - Gastruloid morphometric analysis
  - Fluid phase uptake assay
  - FRET analysis
  - Clonogenicity assay
  - Scanning Electron Microscopy
  - Plasmids
- QUANTIFICATION AND STATISTICAL ANALYSIS

SUPPLEMENTAL INFORMATION

Supplemental Information can be found online at https://doi.org/10.1016/j.stem.2020.10.018.

ACKNOWLEDGMENTS

We thank P. Attlesey and staff for animal husbandry, Carla Mulas for comments on the manuscript, Giorgio Scita for advice on endocytosis, Ortrud Warlick for help with setting up the optical tweezer, the MRC LMCB light microscopy facility for technical support, Anan Ragab and Mark Turmaine for their help with SEM, and members of the Paluch lab (in particular Agathe Chaigne and Murielle Serres) and of the Chalut lab (in particular Carla Mulas and Chibeza Agley) for technical help and advice at different stages of this project. This work was supported by the European Union's Horizon 2020 research and innovation programme under the Marie Sklodowska-Curie grant agreement No 641639 (ITN Biopol, H.D. and E.K.P.), the Medical Research Council UK (MRC programme award MC_UU_12018/5, H.D. and E.K.P.), the Human Frontier Science Program (Young Investigator Grant RGY 66/2013, to E.K.P.), the Leverhulme Trust (Leverhulme Prize in Biological Sciences to E.K.P.), European Research Council (ERC) Consolidator Grants (CellFateTech, 772798, to K.J.C. and NanoMechShape, 820188, to E.K.P.), a core support grant from the Wellcome Trust and Medical Research Council to the Wellcome Trust - Medical Research Council Cambridge Stem Cell Institute (203151/Z/16/Z, K.J.C.).

AUTHOR CONTRIBUTIONS

H.D., E.K.P., and K.J.C. designed the research. E.K.P and K.J.C. supervised the research. H.D. carried out the bulk of the experiments and data analysis. H.D., E.K.P., and K.J.C. wrote the paper. A.S. carried out the gastruloids experiments and analysis. A.Y. carried out the embryo experiments. C.L. carried out the RNA-seq analysis. E.K.P and K.J.C. acquired the funding. P.H.J. built the optical tweezers setup. All of the authors discussed the results and manuscript.

DECLARATION OF INTERESTS

The authors declare no competing interests.

Received: December 3, 2019
Revised: September 18, 2020
Accepted: October 28, 2020
Published: November 19, 2020

REFERENCES

Anders, S., Pyl, P.T., and Huber, W. (2015). HTSeq–a Python framework to work with high-throughput sequencing data. Bioinformatics *31*, 166–169.

Arnold, T.R., Stephenson, R.E., and Miller, A.L. (2017). Rho GTPases and actomyosin: partners in regulating epithelial cell-cell junction structure and function. Exp. Cell Res. *358*, 20–30.

Avior, Y., Sagi, I., and Benvenisty, N. (2016). Pluripotent stem cells in disease modelling and drug discovery. Nat. Rev. Mol. Cell Biol. *17*, 170–182.

Baillie-Johnson, P., van den Brink, S.C., Balayo, T., Turner, D.A., and Martinez Arias, A. (2015). Generation of Aggregates of Mouse Embryonic Stem Cells that Show Symmetry Breaking, Polarization and Emergent Collective Behaviour In Vitro. J. Vis. Exp. (105), 53252.

Bellas, E., and Chen, C.S. (2014). Forms, forces, and stem cell fate. Curr. Opin. Cell Biol. *31*, 92–97.

Bergert, M., Lembo, S., Milovanović, D., Börmel, M., Neveu, P., and Diz-Muñoz, A. (2020). Cell surface mechanics gate stem cell differentiation. Cell Stem Cell *28*, this issue, 209–216.

Biswas, A., Kashyap, P., Datta, S., Sengupta, T., and Sinha, B. (2018). Cholesterol depletion by MβCD enhances membrane tension, its heterogeneity and affects cellular integrity. BioRxiv. https://doi.org/10.1101/264036.

Boroviak, T., Loos, R., Lombard, P., Okahara, J., Behr, R., Sasaki, E., Nichols, J., Smith, A., and Bertone, P. (2015). Lineage-Specific Profiling Delineates the Emergence and Progression of Naive Pluripotency in Mammalian Embryogenesis. Dev. Cell *35*, 366–382.

Chalut, K.J., and Paluch, E.K. (2016). The Actin Cortex: A Bridge between Cell Shape and Function. Dev. Cell *38*, 571–573.

CellPress
OPEN ACCESS

Charras, G., and Paluch, E. (2008). Blebs lead the way: how to migrate without lamellipodia. Nat. Rev. Mol. Cell Biol. *9*, 730–736.

Chen, G., Schell, J.P., Benitez, J.A., Petropoulos, S., Yilmaz, M., Reinius, B., Alekseenko, Z., Shi, L., Hedlund, E., Lanner, F., et al. (2016). Single-cell analyses of X Chromosome inactivation dynamics and pluripotency during differentiation. Genome Res. *26*, 1342–1354.

Chugh, P., Clark, A.G., Smith, M.B., Cassani, D.A.D., Dierkes, K., Ragab, A., Roux, P.P., Charras, G., Salbreux, G., and Paluch, E.K. (2017). Actin cortex architecture regulates cell surface tension. Nat. Cell Biol. *19*, 689–697.

Clark, A.G., Wartlick, O., Salbreux, G., and Paluch, E.K. (2014). Stresses at the cell surface during animal cell morphogenesis. Curr. Biol. *24*, R484–R494.

Connelly, J.T., Gautrot, J.E., Trappmann, B., Tan, D.W.-M., Donati, G., Huck, W.T.S., and Watt, F.M. (2010). Actin and serum response factor transduce physical cues from the microenvironment to regulate epidermal stem cell fate decisions. Nat. Cell Biol. *12*, 711–718.

Cunningham, F., Amode, M.R., Barrell, D., Beal, K., Billis, K., Brent, S., Carvalho-Silva, D., Clapham, P., Coates, G., Fitzgerald, S., et al. (2015). Ensembl 2015. Nucleic Acids Res. *43*, D662–D669.

Dai, J., and Sheetz, M.P. (1995). Regulation of endocytosis, exocytosis, and shape by membrane tension. Cold Spring Harb. Symp. Quant. Biol. *60*, 567–571.

Deathridge, J., Antolović, V., Parsons, M., and Chubb, J.R. (2019). Live imaging of ERK signaling dynamics in differentiating mouse embryonic stem cells. Development *146*, dev172940.

Diz-Muñoz, A., Krieg, M., Bergert, M., Ibarlucea-Benitez, I., Muller, D.J., Paluch, E., and Heisenberg, C.-P. (2010). Control of directed cell migration in vivo by membrane-to-cortex attachment. PLOS Biol. *8*, e1000544.

Diz-Muñoz, A., Fletcher, D.A., and Weiner, O.D. (2013). Use the force: membrane tension as an organizer of cell shape and motility. Trends Cell Biol. *23*, 47–53.

Dutta, D., and Donaldson, J.G. (2012). Search for inhibitors of endocytosis: Intended specificity and unintended consequences. Cell. Logist. *2*, 203–208.

Engler, A.J., Sen, S., Sweeney, H.L., and Discher, D.E. (2006). Matrix elasticity directs stem cell lineage specification. Cell *126*, 677–689.

Evans, M.J., and Kaufman, M.H. (1981). Establishment in culture of pluripotential cells from mouse embryos. Nature *292*, 154–156.

Gautreau, A., Louvard, D., and Arpin, M. (2000). Morphogenic effects of ezrin require a phosphorylation-induced transition from oligomers to monomers at the plasma membrane. J. Cell Biol. *150*, 193–203.

Gudipaty, S.A., Lindblom, J., Loftus, P.D., Redd, M.J., Edes, K., Davey, C.F., Krishnegowda, V., and Rosenblatt, J. (2017). Mechanical stretch triggers rapid epithelial cell division through Piezo1. Nature *543*, 118–121.

Guilak, F., Cohen, D.M., Estes, B.T., Gimble, J.M., Liedtke, W., and Chen, C.S. (2009). Control of stem cell fate by physical interactions with the extracellular matrix. Cell Stem Cell *5*, 17–26.

Halder, G., Dupont, S., and Piccolo, S. (2012). Transduction of mechanical and cytoskeletal cues by YAP and TAZ. Nat. Rev. Mol. Cell Biol. *13*, 591–600.

Heisenberg, C.-P., and Bellaïche, Y. (2013). Forces in tissue morphogenesis and patterning. Cell *153*, 948–962.

Huang, W., Sherman, B.T., and Lempicki, R.A. (2009a). Bioinformatics enrichment tools: paths toward the comprehensive functional analysis of large gene lists. Nucleic Acids Res. *37*, 1–13.

Huang, W., Sherman, B.T., and Lempicki, R.A. (2009b). Systematic and integrative analysis of large gene lists using DAVID bioinformatics resources. Nat. Protoc. *4*, 44–57.

Kalkan, T., Olova, N., Roode, M., Mulas, C., Lee, H.J., Nett, I., Marks, H., Walker, R., Stunnenberg, H.G., Lilley, K.S., et al. (2017). Tracking the embryonic stem cell transition from ground state pluripotency. Development *144*, 1221–1234.

Koser, D.E., Thompson, A.J., Foster, S.K., Dwivedy, A., Pillai, E.K., Sheridan, G.K., Svoboda, H., Viana, M., Costa, L.D., Guck, J., et al. (2016). Mechanosensing is critical for axon growth in the developing brain. Nat. Neurosci. *19*, 1592–1598.

Kunath, T., Saba-El-Leil, M.K., Almousailleakh, M., Wray, J., Meloche, S., and Smith, A. (2007). FGF stimulation of the Erk1/2 signalling cascade triggers transition of pluripotent embryonic stem cells from self-renewal to lineage commitment. Development *134*, 2895–2902.

Lieber, A.D., Yehudai-Resheff, S., Barnhart, E.L., Theriot, J.A., and Keren, K. (2013). Membrane tension in rapidly moving cells is determined by cytoskeletal forces. Curr. Biol. *23*, 1409–1417.

Liu, C., Li, Y., Semenov, M., Han, C., Baeg, G.-H., Tan, Y., Zhang, Z., Lin, X., and He, X. (2002). Control of β-catenin phosphorylation/degradation by a dual-kinase mechanism. Cell *108*, 837–847.

Love, M.I., Huber, W., and Anders, S. (2014). Moderated estimation of fold change and dispersion for RNA-seq data with DESeq2. Genome Biol. *15*, 550.

Matsui, T., Maeda, M., Doi, Y., Yonemura, S., Amano, M., Kaibuchi, K., Tsukita, S., and Tsukita, S. (1998). Rho-kinase phosphorylates COOH-terminal threonines of ezrin/radixin/moesin (ERM) proteins and regulates their head-to-tail association. J. Cell Biol. *140*, 647–657.

McBeath, R., Pirone, D.M., Nelson, C.M., Bhadriraju, K., and Chen, C.S. (2004). Cell shape, cytoskeletal tension, and RhoA regulate stem cell lineage commitment. Dev. Cell *6*, 483–495.

Mendoza, P., Díaz, J., Silva, P., and Torres, V.A. (2014). Rab5 activation as a tumor cell migration switch. Small GTPases *5*, e28195.

Molotkov, A., Mazot, P., Brewer, J.R., Cinalli, R.M., and Soriano, P. (2017). Distinct Requirements for FGFR1 and FGFR2 in Primitive Endoderm Development and Exit from Pluripotency. Dev. Cell *41*, 511–526.e4.

Mulas, C., Kalkan, T., von Meyenn, F., Leitch, H.G., Nichols, J., and Smith, A. (2019). Defined conditions for propagation and manipulation of mouse embryonic stem cells. Development *146*, dev173146.

Murray, P., Prewitz, M., Hopp, I., Wells, N., Zhang, H., Cooper, A., Parry, K.L., Short, R., Antoine, D.J., and Edgar, D. (2013). The self-renewal of mouse embryonic stem cells is regulated by cell-substratum adhesion and cell spreading. Int. J. Biochem. Cell Biol. *45*, 2698–2705.

Nambiar, R., McConnell, R.E., and Tyska, M.J. (2009). Control of cell membrane tension by myosin-I. Proc. Natl. Acad. Sci. USA *106*, 11972–11977.

Nett, I.R., Mulas, C., Gatto, L., Lilley, K.S., and Smith, A. (2018). Negative feedback via RSK modulates Erk-dependent progression from naïve pluripotency. EMBO Rep. *19*, 45642.

Nichols, J., Silva, J., Roode, M., and Smith, A. (2009). Suppression of Erk signalling promotes ground state pluripotency in the mouse embryo. Development *136*, 3215–3222.

Palamidessi, A., Malinverno, C., Frittoli, E., Corallino, S., Barbieri, E., Sigismund, S., Beznoussenko, G.V., Martini, E., Garre, M., Ferrara, I., et al. (2019). Unjamming overcomes kinetic and proliferation arrest in terminally differentiated cells and promotes collective motility of carcinoma. Nat. Mater. *18*, 1252–1263.

Paluch, E., and Heisenberg, C.-P. (2009). Biology and physics of cell shape changes in development. Curr. Biol. *19*, R790–R799.

Pepe-Mooney, B.J., Dill, M.T., Alemany, A., Ordovas-Montanes, J., Matsushita, Y., Rao, A., Sen, A., Miyazaki, M., Anakk, S., Dawson, P.A., et al. (2019). Single-Cell Analysis of the Liver Epithelium Reveals Dynamic Heterogeneity and an Essential Role for YAP in Homeostasis and Regeneration. Cell Stem Cell *25*, 23–38.e8.

Pertz, O., Hodgson, L., Klemke, R.L., and Hahn, K.M. (2006). Spatiotemporal dynamics of RhoA activity in migrating cells. Nature *440*, 1069–1072.

Platta, H.W., and Stenmark, H. (2011). Endocytosis and signaling. Curr. Opin. Cell Biol. *23*, 393–403.

Pontes, B., Monzo, P., and Gauthier, N.C. (2017a). Membrane tension: a challenging but universal physical parameter in cell biology. Semin. Cell Dev. Biol. *71*, 30–41.

Pontes, B., Monzo, P., Gole, L., Le Roux, A.-L., Kosmalska, A.J., Tam, Z.Y., Luo, W., Kan, S., Viasnoff, V., Roca-Cusachs, P., et al. (2017b). Membrane tension controls adhesion positioning at the leading edge of cells. J. Cell Biol. *216*, 2959–2977.

Pouille, P.-A., Ahmadi, P., Brunet, A.-C., and Farge, E. (2009). Mechanical signals trigger Myosin II redistribution and mesoderm invagination in Drosophila embryos. Sci. Signal. *2*, ra16.

Przybyla, L., Lakins, J.N., and Weaver, V.M. (2016). Tissue Mechanics Orchestrate Wnt-Dependent Human Embryonic Stem Cell Differentiation. Cell Stem Cell *19*, 462–475.

Rappaport, J., Manthe, R.L., Solomon, M., Garnacho, C., and Muro, S. (2016). A Comparative Study on the Alterations of Endocytic Pathways in Multiple Lysosomal Storage Disorders. Mol. Pharm. *13*, 357–368.

Rauch, C., Brunet, A.-C., Deleule, J., and Farge, E. (2002). C2C12 myoblast/osteoblast transdifferentiation steps enhanced by epigenetic inhibition of BMP2 endocytosis. Am. J. Physiol. Cell Physiol. *283*, C235–C243.

Raucher, D., and Sheetz, M.P. (2000). Cell spreading and lamellipodial extension rate is regulated by membrane tension. J. Cell Biol. *148*, 127–136.

Razzell, W., Wood, W., and Martin, P. (2014). Recapitulation of morphogenetic cell shape changes enables wound re-epithelialisation. Development *141*, 1814–1820.

Riedl, J., Flynn, K.C., Raducanu, A., Gärtner, F., Beck, G., Bösl, M., Bradke, F., Massberg, S., Aszodi, A., Sixt, M., and Wedlich-Söldner, R. (2010). Lifeact mice for studying F-actin dynamics. Nat. Methods *7*, 168–169.

Rouven Brückner, B., Pietuch, A., Nehls, S., Rother, J., and Janshoff, A. (2015). Ezrin is a Major Regulator of Membrane Tension in Epithelial Cells. Sci. Rep. *5*, 14700.

Schindelin, J., Arganda-Carreras, I., Frise, E., Kaynig, V., Longair, M., Pietzsch, T., Preibisch, S., Rueden, C., Saalfeld, S., Schmid, B., et al. (2012). Fiji: an open-source platform for biological-image analysis. Nat. Methods *9*, 676–682.

Segel, M., Neumann, B., Hill, M.F.E., Weber, I.P., Viscomi, C., Zhao, C., Young, A., Agley, C.C., Thompson, A.J., Gonzalez, G.A., et al. (2019). Niche stiffness underlies the ageing of central nervous system progenitor cells. Nature *573*, 130–134. https://www.ncbi.nlm.nih.gov/entrez/eutils/elink.fcgi?dbfrom=pubmed&retmode=ref&cmd=prlinks&id=31413369.

Solis, A.G., Bielecki, P., Steach, H.R., Sharma, L., Harman, C.C.D., Yun, S., de Zoete, M.R., Warnock, J.N., To, S.D.F., York, A.G., et al. (2019). Mechanosensation of cyclical force by PIEZO1 is essential for innate immunity. Nature *573*, 69–74.

Solter, D., and Knowles, B.B. (1975). Immunosurgery of mouse blastocyst. Proc. Natl. Acad. Sci. USA *72*, 5099–5102.

Sorkin, A., and von Zastrow, M. (2009). Endocytosis and signalling: intertwining molecular networks. Nat. Rev. Mol. Cell Biol. *10*, 609–622.

Tee, S.-Y., Fu, J., Chen, C.S., and Janmey, P.A. (2011). Cell shape and substrate rigidity both regulate cell stiffness. Biophys. J. *100*, L25–L27.

Thottacherry, J.J., Kosmalska, A.J., Kumar, A., Vishen, A.S., Elosegui-Artola, A., Pradhan, S., Sharma, S., Singh, P.P., Guadamillas, M.C., Chaudhary, N., et al. (2018). Mechanochemical feedback control of dynamin independent endocytosis modulates membrane tension in adherent cells. Nat. Commun. *9*, 4217.

Totaro, A., Castellan, M., Battilana, G., Zanconato, F., Azzolin, L., Giulitti, S., Cordenonsi, M., and Piccolo, S. (2017). YAP/TAZ link cell mechanics to Notch signalling to control epidermal stem cell fate. Nat. Commun. *8*, 15206.

Turner, D.A., Girgin, M., Alonso-Crisostomo, L., Trivedi, V., Baillie-Johnson, P., Glodowski, C.R., Hayward, P.C., Collignon, J., Gustavsen, C., Serup, P., et al. (2017). Anteroposterior polarity and elongation in the absence of extra-embryonic tissues and of spatially localised signalling in gastruloids: mammalian embryonic organoids. Development *144*, 3894–3906.

Verstreken, C.M., Labouesse, C., Agley, C.C., and Chalut, K.J. (2019). Embryonic stem cells become mechanoresponsive upon exit from ground state of pluripotency. Open Biol. *9*, 180203.

Wray, J., Kalkan, T., Gomez-Lopez, S., Eckardt, D., Cook, A., Kemler, R., and Smith, A. (2011). Inhibition of glycogen synthase kinase-3 alleviates Tcf3 repression of the pluripotency network and increases embryonic stem cell resistance to differentiation. Nat. Cell Biol. *13*, 838–845.

Wu, T.D., and Nacu, S. (2010). Fast and SNP-tolerant detection of complex variants and splicing in short reads. Bioinformatics *26*, 873–881.

Yanagida, A., Revell, C., Stirparo, G.G., Corujo-Simon, E., Aspalter, I.M., Peters, R., Belly, H.D., Cassani, D.A.D., Achouri, S., Blumenfeld, R., et al. (2020). Cell surface fluctuations regulate early embryonic lineage sorting. BioRxiv. https://doi.org/10.1101/2020.08.16.250084.

Ying, Q.-L., Wray, J., Nichols, J., Batlle-Morera, L., Doble, B., Woodgett, J., Cohen, P., and Smith, A. (2008). The ground state of embryonic stem cell self-renewal. Nature *453*, 519–523.

Yui, S., Azzolin, L., Maimets, M., Pedersen, M.T., Fordham, R.P., Hansen, S.L., Larsen, H.L., Guiu, J., Alves, M.R.P., Rundsten, C.F., et al. (2018). YAP/TAZ-Dependent Reprogramming of Colonic Epithelium Links ECM Remodeling to Tissue Regeneration. Cell Stem Cell *22*, 35–49.e7.

CellPress
OPEN ACCESS

STAR★METHODS

KEY RESOURCES TABLE

REAGENT or RESOURCE	SOURCE	IDENTIFIER
Antibodies		
phospho ERK	Cell Signaling	4370 S
ERK	Cell Signaling	9107 S
Nanog	eBioscience	eBioMLC-51
Otx2	R&D Systems	AF1979
ERM	Cell Signaling	3142S
Phospho ERM	Cell Signaling	3149S
GAPDH	Abcam	ab8245
His3	Abcam	Ab1701
Fgfr1	Abcam	ab10646
Goat anti-Rat IgG, Secondary Antibody, Alexa Fluor 680	Thermofischer Scientific	A32787
Donkey anti-Goat IgG , Secondary Antibody, Alexa Fluor 488	Thermofischer Scientific	A-11055
Chemicals, Peptides, and Recombinant Proteins		
B27	Life technologies	Cat#12587010
CHIRON	Cambridge Bioscience	Cat#CAY13122
PD 0325901	Sigma-Aldrich	Cat#PZ0162
LIF	Merck Millipore	Cat# ESG1107
Insulin zinc	Sigma-Aldrich	Cat#I9278
Apotransferrin	Sigma-Aldrich	Cat# T1147
Laminin	Sigma-Aldrich	Cat#11243217001
Lipofectamin^{TM} RNAimax	Thermofischer Scientific	Cat# 13778075
Accutase	Sigma-Aldrich	Cat#A6964
DMEM/F-12, 1:1 mixture	Sigma-Aldrich	Cat#D6421-6
Neurobasal medium	Life technologies	Cat#21103-049
Lipofectamine® 2000 Transfection Reagent	Life technologies	Cat#11668-027
Hoechst	ThermoFischer	Cat#34580
DAPI	Sigma-Aldrich	Cat#9542
Dynasore	Sigma-Aldrich	Cat#D7693
NSC 23766	Tocris	Cat#2161
Methyl-B-Cyclodextrin	Sigma-Aldrich	Cat#C4555
BI-D1870	VWR	Cat#501437-28-1
PitStop2	Abcam	Cat#Ab120687
Chlorpromazine hydrochloride	Sigma-Aldrich	Cat#D22914
Dextran, Alexa Fluor 647, 10,000 MW	ThermoFischer	Cat#D22914
pHrodo Red Dextran, 10,000 MW	ThermoFischer	Cat#P10361
Critical Commercial Assays		
Active RhoA pulldown assay	Cytoskeleton Inc	Cat#BK036
Deposited Data		
RNA seq: GEO accession number GEO: GSE159433	https://www.ncbi.nlm.nih.gov/geo/query/acc.cgi?acc=GSE159433	N/A
Experimental Models: Cell Lines		
Mouse embryonic stem cells: E14	Chalut lab (Cambridge Stem cell Institute, Cambridge, UK)	N/A
Mouse embryonic stem cells: EZR_CA	Chalut lab (Cambridge Stem cell Institute, Cambridge, UK)	Yanagida et al., 2020

(*Continued on next page*)

Continued		
REAGENT or RESOURCE	SOURCE	IDENTIFIER
Mouse embryonic stem cells: RhoA_CA	Chalut lab (Cambridge Stem cell Institute, Cambridge, UK)	Yanagida et al., 2020
Mouse embryonic stem cells: H2B-mCherry	Nichol's lab (Cambridge Stem cell Institute, Cambridge, UK)	N/A
Mouse embryonic stem cells: LifeAct GFP	Ian Rosewell (Francis Crick Institute, London, UK)	Riedl et al., 2010
Mouse embryonic stem cells: Beta-Catenin KO	Smith's lab (Cambridge Stem cell Institute, Cambridge, UK)	Wray et al., 2011
Oligonucleotides		
SMARTpool: ON-TARGETplus Ezr siRNA	Dharmacon	L-046568-01
SMARTpool: ON-TARGETplus Rdx siRNA	Dharmacon	L-047230-01
SMARTpool: ON-TARGETplus Msn siRNA	Dharmacon	L-044428-01
SMARTpool: ON-TARGETplus MYO1C siRNA	Dharmacon	L-015121-00
SMARTpool: ON-TARGETplus Myo1b siRNA	Dharmacon	L-045103-01
siGENOME Non-Targeting Control siRNA #3	Dharmacon	D-001210-03
siGENOME Non-Targeting Control siRNA #5	Dharmacon	D-001210-05
siGENOME Non-Targeting Control siRNA #2	Dharmacon	D-001210-02
Software and Algorithms		
Fiji	Schindelin et al. (2012)	N/A
Prism 8	Graphpad software, Inc	N/A

RESOURCE AVAILABILITY

Lead Contact

Further information and requests for resources and reagents should be directed to and will be fulfilled by the Lead Contact, Kevin J Chalut, kc370@cam.ac.uk.

Materials Availability

This study did not generate new unique reagents

Data and Code Availability

The accession number for the RNA sequencing data reported in this paper is GEO: GSE159433 (accessible through: https://www.ncbi.nlm.nih.gov/geo/query/acc.cgi?acc=GSE159433)

EXPERIMENTAL MODEL AND SUBJECT DETAILS

Cell lines

ES-E14TG2a (referred as WT) directly derived from mouse embryos were a kind gift from the Austin Smith's laboratory (Cambridge Stem Cell Institute). The LifeAct GFP cell line is from Riedl et al. (2010). These cells have been generated and kindly given to us by Ian Rosewell (Francis Crick Institute, under Holger Gerhardts project license). Dox-inducible IRES mCherry Ezrin_CA & RhoA_CA ES (Yanagida et al., 2020) cells were kindly given to us by Ayaka Yanagida from the Chalut and Nichols's lab. The EZR_CA was engineered using a PiggyBac system with E14 ES cells. The H2B-mCherry cells were kindly given to us by Tim Lohoff from the Nichol and Reik's lab.The β-Catenin Knockout cell line was kindly given to us by the lab of Austin Smith and has been described in Wray et al. (2011).

Mice and mice embryos

Mice used were intercrosses of CD1 (Charles River). All embryos used in this study were obtained from natural mating. Embryo staging was based on the assumption that, on average, mating occurred at midnight so that at midday, the embryos were assigned E0.5. Embryos were flushed at the relevant stages from oviduct (eight-cell stage embryos) using flushing and holding a medium (M2, Sigma). The sex of embryos and the ages of mice using mating were not concerned in this study. This research has been regulated under the Animals (Scientific Procedures) Act 1986 Amendment Regulations 2012 following ethical review by the University of Cambridge Animal Welfare and Ethical Review Body (AWERB). Use of animals in this project was approved by the ethical review committee for the University of Cambridge, and relevant Home Office licenses (Project license No. P76777883) are in place.

METHOD DETAILS

Cell culture, transfections and exit from naive pluripotency

Mouse embryonic stem cells were cultured on Falcon flasks coated with 0.1% gelatine. On occasion, laminin (Sigma-Aldrich #11243217001) coated dishes were used instead of gelatine. ES cells used were E14TG2a. Cells were cultured in N2B27+2i+LIF (2i+L) (Mulas et al., 2019) at 37°C with 7% CO_2. Cells were passaged every other day using Accutase (Sigma-Aldrich, #A6964) and regularly tested for mycoplasma. Culture medium was changed every 24h. The culture medium was made using DMEM/F-12, 1:1 mixture (Sigma-Aldrich, #D6421-6), Neurobasal medium (Life technologies #21103-049), 2.2 mM L-Glutamin, B27 (Life technologies #12587010), 3 μM Chiron (Cambridge Bioscience #CAY13122), 1 μM PD0325901 (Sigma-Aldrich #PZ0162), 20 ng/ml of LIF (Merck Millipore # ESG1107), 50 mM β-Mercapto-ethanol, 12.5 $ng.mL^{-1}$ Insulin zinc (Sigma-Aldrich #I9278) and home-made N2. The 200 X home-made N2 was made using 0.791 $mg.mL^{-1}$ Apotransferrin (Sigma-Aldrich #T1147), 1.688 $mg.mL^{-1}$ Putrescine (Sigma-Aldrich #P5780), 3 μM Sodium Selenite (Sigma-Aldrich #S5261), 2.08 $\mu g.mL^{-1}$ Progesterone (Sigma-Aldrich #P8783), 8.8% BSA. Exit from naive pluripotency was triggered by passaging ~500 000 cells and seeding them in N2B27 only (N2B27) on T25 gelatine coated flasks.

Transfections of plasmids were performed using 5 μg of plasmid and 3.5 μL of Lipofectamine 2000 (ThermoFischer Scientific #11668019), incubated in 250 μL OptiMEM for 5 minutes, then mixed and incubated at room temperature for 30 minutes, and added to cells passaged onto Ibidi dishes with polymer coverslip bottom for observations (IBIDI Scientific, 81156).

Due to the lack of fluorescent tag, transfection efficiency of Rab5a was assessed by performing immunofluorescence against Rab5a of un-transfected and transfected cells. Average fluorescence level was determined in transfected and untransfected cells by measuring average fluorescence intensity using Fiji (Schindelin et al., 2012). Cells with an average fluorescence higher than 20% of the average of untrasnfected cells were counted as being positively transfected. Using this method, we estimate Rab5a transfection efficiency to be ~72%. siRNA transfections were performed using 30 μM of siRNA and 5 μL of Lipofectamine RNAiMAX (ThermoFischer Scientific #13778075) incubated in 250 μL OptiMEM for 5 minutes, then mixed and incubated at room temperature for 30 minutes and added to cells passaged onto micropatterns.

Tether pulling and trap force measurements

Trap force measurements were performed using a home-built optical tweezer using a 4W 1064 nm Laser Quantum Ventus with a 100x oil immersion objective (NA 1.30, CFI Plan Fluor DLL, Nikon) on an inverted microscope (Nikon Eclipse TE2000-U) equipped with a motorized stage (PRIOR Proscan). The optical tweezer was calibrated following Lieber et al. (2013). Measurements were performed using concanavalin-A coated (50 μg/ml) carboxyl latex beads (1.9μm diameter, Thermo Fisher C37278). Beads were incubated on a shaker with concanavalin-A for one hour prior to the experiment. Bead position was recorded every 90 ms in bright field prior and during tether formation. The trap force was calculated based on the calibration of the trap and the bead position using a home-made Fiji (Schindelin et al., 2012) plugin (HDB) (typical values for the trap stiffness were k~0.130 pN/nm.)

RNA extraction and qPCR

Cells were seeded in 6 well dishes in different conditions (N2B27 or 2i+L) and cultured for different periods of time, as indicated. RNA was extracted using a QIAGEN (205310) kit. Extracted RNA cDNA was generated from the RNA according to kit's instructions (Thermo Fischer Scientific #4368814). For the RT-qPCR, pre-designed primers (see table of primers used), and the SYBR Green Master Mix (QIAGEN; 204141) were combined according to the kit's instructions. qPCR was performed on Bio-Rad CFX qPCR.

Western blots

Cells were plated into 6-well plates in different conditions (with or without 2i+L), as indicated. Proteins were extracted on ice using Laemmli buffer (Bio-Rad #1610747). Samples were put at 95°C for 5 min and then sonicated on ice. Isolated whole protein content was measured using the Thermo Scientific Pierce 660nm Protein assay (#22660) using a BSA gradient kit (Bio-Rad; 500-0206) and 20 μg of protein extract was loaded onto either NuPAGE 4%–12% Bis-Tris protein gels (Thermo Fischer Scientific #NP0321BOX) or Novex WedgeWell 14% Tris-Glycine Mini Gels (Thermo Fischer Scientific #XP00140BOX) depending on the size of the protein of interest. Gels were run at 125 V for 2 h. Proteins were transferred for 70 minutes at 70 V to a nitrocellulose membrane (Thermo Fischer Scientific #88024). Membranes were blocked for 90 min using Odyssey blocking buffer (Li-Cor #927-50003). Primary antibodies were added at the proper dilution Table S1 in either TBS-T + 5% milk or 5% BSA and incubated overnight at 4 degrees. Membranes were washed three times for 15 min in TBS with TWEEN. Infrared species-appropriate secondary antibodies were then added Table S1 for one hour. Membranes were washed three times with PBS-T for 15 min and imaged on the Li-Cor Odyssey scanner. Bands were quantified using LI-COR Biosciences Image Studio Lite.

Immunofluorescence

Cells were fixed in IBIDI dishes (IBIDI Scientific, 81156) with 4% formaldehyde. Cells were permeabilized during 10 min in PBS+0.1% Triton-X. Blocking was then done using with 2% FBS 2% BSA in PBS with 0.1% Triton for 45 min. Cells were then incubated for 1h30min with the primary antibody diluted at the appropriate concentration in the same buffer as used for blocking. Cells were then washed 3 times 5 min with PBS+0.1% Triton. Secondary antibodies were added for 1 h diluted at 1:800 in the same buffer

as used for blocking and primary antibody incubation. Cells were washed with PBS + 0.1% Triton 3 times for 5 min. Finally, cells were incubated for 5 min with PBS+DAPI before a final rinse in PBS.

RNA sequencing, data processing, differential expression analysis and gene ontology analysis

Library preparation was done by in-house facility us NEBNext® Multiplex Oligos for Illumina. Paired-end sequencing was performed using the Novaseq sequencing platform, yielding 517 million reads (single-lane). Reads were trimmed and adapters removed using FastQC and TrimGalore (Babraham Bioinformatics). Mouse genome build GRCm38/mm10 was used to align reads with GSNAP version 2015-09-29 (Wu and Nacu, 2010). Genes were annotated using Ensembl release 81 (Cunningham et al., 2015) and read counts were quantified using HTSeq (Anders et al., 2015). Differential expression analysis was computed using DESeq2 (Love et al., 2014) a design containing an interaction term (Time point:CellType).

For gene ontology analysis, the significant interaction terms at 24hrs and 48hrs were selected (i.e., differently regulated between iEZR_CA cells and wild-type celld during exit from naive pluripotency), using thresholds of base mean > 5 and an absolute log2 fold change > 1. DAVID 6.8 (Huang et al., 2009a, 2009b) was used to compute the statistical enrichment of Gene Ontology terms. For each category (KEGG Pathway and Cellular Component), the list of enriched terms was filtered using Benjamini-Hochberg false-discovery rate correction (p_adj < 0.1). Additionally, only terms with fold enrichment > = 2 and at least 1% of annotated genes regulated were selected. The top 5 (ordered on increasing adjusted p value) are plotted for each of the four functional annotations category.

Highly variable genes were determined by averaging, for each gene, the intra-group variance (3 replicates per group, 6 experimental groups). The intra-group (or technical) variance was then compared to the distribution of variance computed over 3 randomly selected samples (inter-group variance). Highly variable genes were those for which the technical variance was significantly lower than the inter-group variance.

Micropatterning

Glass coverslips (Marienfekd GmBH) were sonicated in 1 M HCl for 5-15 minutes, washed and plasma-treated for 30 s for glass passivation. Then 0.1 mg/mL of PLL-g-PEG (PLL(20)-g[3.5]-PEG(2)/Atto663 from SuSoS) solution was added to the coverslips for 30 min at room temperature. Chrome MASK from Delta Mask was used. The mask was illuminated with UV light for 5 min on the chrome side, then the PEG-coverslips were added on the chrome side and illuminated for 6 min to burn the PEG in the shape of the desired pattern (circles in our case). Coverslips were then dried and stored. Coverslips were rehydrated before use.

For immunofluorescence assays, we seeded the cells onto the patterns (coated with laminin, see above for details) in 2i+L at a density allowing for the formation of small colonies, occupying the whole available space on the small circles while still having free space on the bigger circles. Cells were then washed three times in PBS and placed in N2B27 only without 2i+L. After 24h in N2B27, cells were fixed and immunostained.

Generation of chimeras

ES cells (five to eight cells per embryo) were injected into eight-cells embryos via a laser-generated perforation in the zona pellucida using XYClone (Hamilton Thorne Biosciences). Injected embryos were cultured in N2B27 with or without 1 μg/ml Dox for two-days for eight-cells injection or one-day for blastocyst injection, an equivalent of E4.5 blastocysts at 37°C and 5% CO_2.

Isolation of ICMs from embryos, and *in vitro* culture

Embryo and cell manipulations were carried out under a dissecting microscope (Leica Microsystems). The zona pellucida was removed using acid Tyrode's solution (Sigma) at E4.0. E4.5 blastocysts were subjected to immunosurgery as previously described (Solter and Knowles, 1975). In brief, blastocysts were incubated for 45-60 minutes in a 1:5 dilution of anti-mouse rabbit serum (Sigma) in N2B27, washed in N2B27 and further incubated for 30-60 minutes in a 1:5 dilution of rat serum (in-house) in N2B27 for the complement reaction. The ICM was subsequently cleaned from residual trophectoderm with a narrowly fitting glass pipette. Isolated ICMs were culture in N2B27 at 37°C and 5% CO_2 with or without 1 μg/ml Dox an equivalent to E5.5 in which PrE lineage cells surround the matured EPI.

RhoA pulldown assay

RhoA pulldown assay was performed using the Cytoskeleton Inc pull down kit (#BK036) and carefully following the provided instructions. In a nutshell, the assay is using beads coated with the Rho binding domain of Rhotekin, a rho effector protein which bind specifically to the GTP-bound RhoA. His tagged RhoA protein and GTPγS were used as controls.

Immunofluorescence staining

Embryos or one-day cultured isolate ICMs were fixed with 4% paraformaldehyde (PFA; Thermo Fisher Scientific) in Phosphate buffered saline (PBS; Sigma) at room temperature for 15 minutes. Then, the samples were rinsed in PBS containing 3 mg/ml polyvinylpyrrolidone (PBS/PVP; Sigma), permeabilised with PBS/PVP containing 0.25% Triton X-100 for 30 minutes. Blocking was performed with a buffer comprising PBS containing 0.1% bovine serum albumin (BSA; Sigma), 0.01%Twenn20 (Sigma) and 2% donkey serum at 4°C for 2-3 hours. Primary antibodies (See Table S1) were diluted in blocking buffer, and samples were incubated in the appropriate antibody solution at 4°C overnight. They were rinsed three times in blocking buffer for 15 minutes ~each. Secondary antibodies were diluted in blocking buffer with 500 ng/ml 4',6-diamidino-2-phenylindole (DAPI; Invitrogen) and samples

were incubated in the appropriate antibody solution at room temperature for one hour in the dark. They were rinsed three times in blocking buffer for 15 minutes ~each. Whole staining process was performed on a microwell mini Tray (Nunc). Embryos or cultured isolated ICMs were put in a small drop of blocking buffer on poly-D-lysine (Sigma) coated glass-bottom dishes (MatTek) covered with mineral oil (Sigma), and their images were acquired using a Leica TCS SP5 confocal microscope.

Gastruloid culture

Gastruloids produced as described before (Baillie-Johnson et al., 2015). Briefly, mouse ES cells were detached from gelatin-coated vessels using accutase for 4 min (Sigma, A6964). The iEZR_CA ES cells were cultured with doxycycline for 24 h or 48 h before aggregation and during the following protocol. Detached cells were then washed once with warm N2B27 and twice with warm PBS (Sigma, D8537). In between the washes, the cells were pelleted for 3 min at 170 x g. Subsequently, the cells were resuspended to fresh N2B27 and the number of cells counted using an automated cell counter (Merck, Scepter 2.0, PHCC20040). The cell suspension was diluted so that 40 ul of suspension had 300 cells. 40 ul of suspension was then transferred to low attachment 96 well round-bottomed plates (Sigma, M3562) for 48 h of aggregation. Next, 150 ul of fresh N2B27 medium with 3 uM CHRI99021 (Cambridge Bioscience, CAY13122) was pipetted to the wells for 24 hours. After which 150 ul of fresh N2B27 medium was changed to the wells daily until 120 hours.

Gastruloid morphometric analysis

WT and iEZR_CA organoids were imaged using a cell culture microscope (Olympus, CKX53SF) and 10x air objective (Olympus, NA 0.25) mounted with a digital camera (Canon, EOS 250D). The resulting images were analyzed with ImageJ Fiji distribution (Schindelin et al., 2012). Briefly, the images were opened as an image stack and converted to 8-bit grayscale images. Next, the grayscale images were thresholded manually to best segment the organoid outlines. The resulted binary masks were then measured using Fiji build in analyze particles function. ‘‘Feret’s diameter’’ and ‘‘shape descriptors’’ were recorded and analyzed.

Fluid phase uptake assay

Cells were seeded on IBIDI dishes (IBIDI Scientific, 81156) a few hours before imaging. Cells were then incubated for 10 min in N2B27 or 2i+L with 10 μg of either pH-Rhodo Dextran (pHrodo Red Dextran, 10,000 MW, for Endocytosis, Thermofischer Scientific) or Alexa Fluor Dextran (Dextran, Alexa Fluor 647; 10,000 MW, Thermofischer Scientific) (Rappaport et al., 2016). Cells were then rinsed immediately prior to imaging. Each dish was imaged for 30 min.

For quantifications with either pH Rhodo or Regular Dextran, total intensity inside the cell was measured with Fiji by using a sum z projection. The signal was corrected by taking a ROI of the background of a similar size as of the measured cells. The background signal was then subtracted from the signal from cell. Only single cells or groups of cells with clear boundaries were quantified. CellMask Deep Red (Invitrogen # C10046) was used to identify individual cell boundaries. Experiments were always performed side by side with cells in 2i+LIF as control. Data are normalized to cells in 2i+L.

FRET analysis

Imaging was performed on an inverted confocal microscope (Olympus FV1200), at 37°C with 5% CO_2 for live imaging, taking z stacks 10 μm in total height with a 1 μm step using a 63x objective. For FRET quantifications, we used a home-written (HDB) custom Macro in Fiji (Schindelin et al., 2012) FRET and donor channel signals were corrected for background.

For the FRET sensor for RhoA activation (Pertz et al., 2006), a sum-projection was performed before FRET quantification. We then measured average ratio in each cell by using three circular ROIs (of 1 μm diameter) in the region of positive FRET ratio (thus at the cell cytoplasm or cortex and not in the nucleus for example). We then averaged the obtained data for each cell.

For the FRET sensor for ERK activation in the endosome, vesicles were semi-automatically identified and average fluorescence was quantified in each vesicle across all the channels. Small Z maximum projections (over 3 z slices of 1 μm) were quantified in order to ensure capture entire endocytotic vesicles. We then calculated the ratio of values obtained in donor and FRET channels to obtain FRET ratio following Deathridge et al. (2019) and Palamidessi et al. (2019) methods.

Clonogenicity assay

To test for speed and efficiency of exit from pluripotency, replating assays were performed. For each experimental condition, ~400 000 cells were plated onto 6-well dishes coated with 0.1% gelatin, in N2B27 to induce exit from naive pluripotency. After 48h in N2B27, the cells were resuspended and counted. A specific number of cells (typically 400) was then replated in N2B27+2i+L onto a 12-well plate. After 4 days, the number of colonies was manually counted; as only naive cells survive in 2i+L, this assay quantifies the efficiency of pluripotency exit. As a positive control, cells cultured in 2i+L were replated in 2i+L. Occasionally replated cells were tested for naive pluripotency using a Blue-color AP staining kit to add another control that replated cells were indeed naive pluripotent (Cambridge Bioscience #AP100B-1).

Scanning Electron Microscopy

Samples were fixed with glutaraldehyde 1%. Preparation for scanning electron microscopy was performed as in Chugh et al. (2017) without membrane extraction. Jeol 7401 - high resolution Field Emission Scanning Electron Microscope was used for imaging.

Plasmids

Rab5a and FRET plasmids were provided by Giorgio Scita's lab (Palamidessi et al., 2019)

Ezr_GFP and EZR_CA_GFP were provided by Guillaume Charras (Gautreau et al., 2000)

FRET RhoA sensor was obtained from Adgene (#12150) and is characterized in Pertz et al. (2006))

QUANTIFICATION AND STATISTICAL ANALYSIS

Live imaging was performed on an inverted confocal microscope (Olympus FV1200), at 37°C with 5% CO_2, taking z stacks 10 μm in total height with a 1 μm step using a 63x objective and using low laser power. Fix imaging was performed on an inverted confocal microscope (Leica TCS SP5) taking z stacks 20 μm in total height with a 1 μm step using a 63x objective .All image analysis were performed using Fiji (Schindelin et al., 2012).

To measure Nanog and Otx2 levels, we used DAPI to locate the cell nuclei, identified the nuclei mid plane and make a ROI of the nucleus. We then used this ROI to measure average Nanog or Otx2 intensity per nucleus, thus controlling for cell/nuclear size in measuring transcription factor expression.

For pERM measurements by IF, we used a sum-projection. We then identified ROIs in the cell (using the cortical actin identified via the phalloidin channel (Alexa Fluor 568 Phalloidin (ThermoFischer Scientific # A12380)) as boundary for the ROI) and measured the integrated signal (the mean intensity signal*number of pixels in the selection) to compensate for differences in cell size. Additionally, we corrected the measured values by subtracting background using the same ROI as for the cell but in a region with no cell present. Data were normalized to round cells. signal was used to determine cell boundaries and to classify cells according to shape based on visual inspection.

For Fgfr1 quantifications, average fluorescence was measured in each cell using CellMask Deep Red (Invitrogen # C10046) as a marker to locate individual cells. Average intensity was then manually measured in the midplane of the cells by taking a ROI following the cell boundary.

For #blebs in Figure S1, the number of blebs was manually counted at every time point (every 20 minutes) across a z stack using LifeAct and brightfield as guides to identify individual blebs (note that only actin filled blebs could be observed with this method).

For all statistical analysis, PRISM 7 (Graphpad software, Inc) was used. Statistical details can be found in the legend of each figure. D'agostino and pearson test was used to test for normality. N represents number of independent biological replicates. Pooled independent experiments are used in dot plots.

Cell Stem Cell, Volume *28*

Supplemental Information

Membrane Tension Gates ERK-Mediated

Regulation of Pluripotent Cell Fate

Henry De Belly, Aki Stubb, Ayaka Yanagida, Céline Labouesse, Philip H. Jones, Ewa K. Paluch, and Kevin J. Chalut

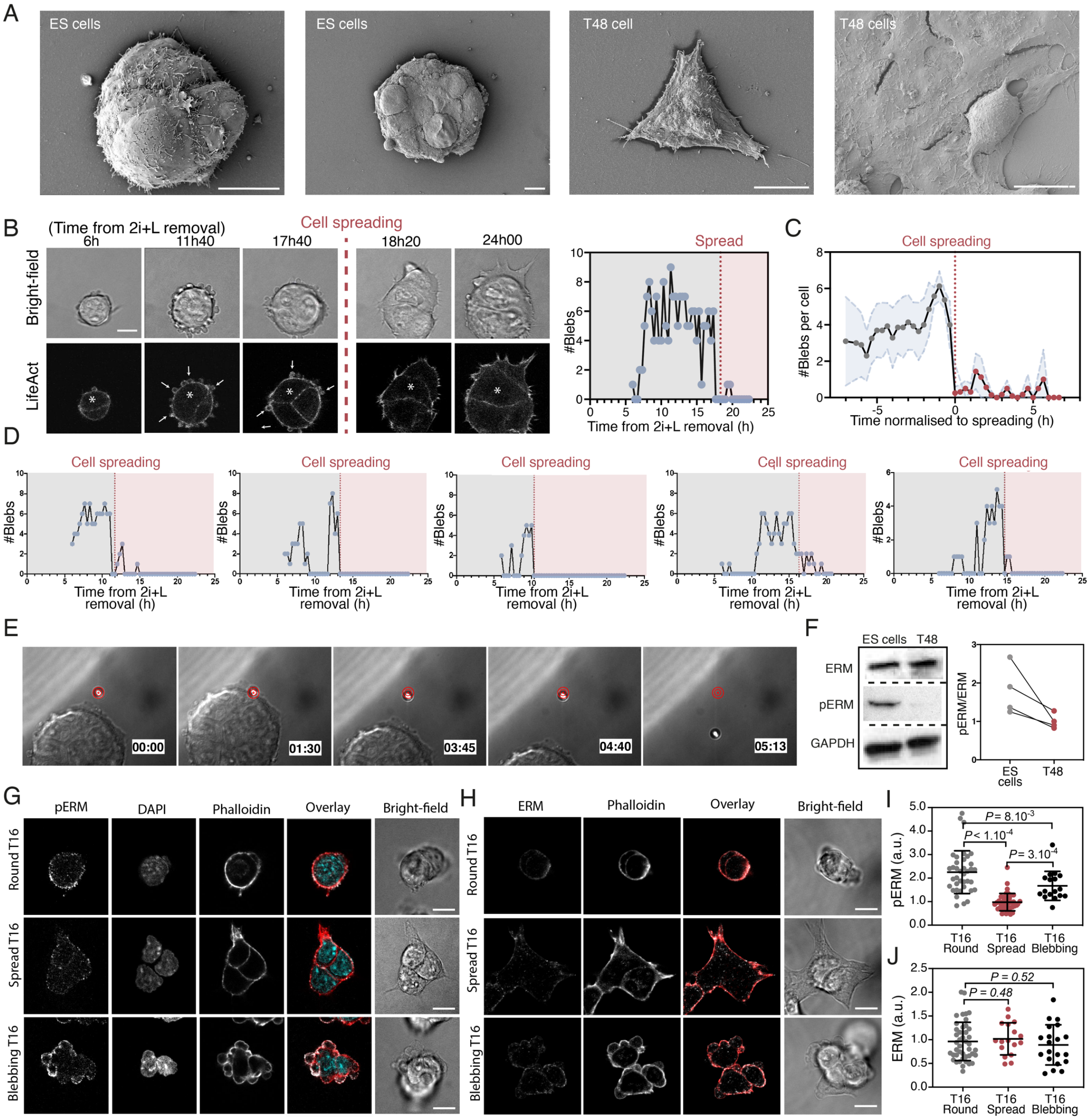

Figure S1: Membrane to cortex attachment and membrane tension are decreased during early differentiation (related to Figure 1). A, Representative SEM images of ES and T48 cells. Scale bars, 10 µm. **B,** Left: time-lapse of representative ES cells expressing LifeAct-GFP and exiting naïve pluripotency. White arrows: cellular blebs. Red dotted line indicates approximate time of cell spreading. Right: quantification of the number of blebs in the cell marked with a white star. **C,** Quantification of cell blebbing during exit from naïve pluripotency. Time is normalised to the time of spreading (mean ± SD, n = 20, N=2). **D**, Quantification of blebbing in six different ES cells expressing LifeAct-GFP during exiting naïve pluripotency. Red dotted line indicates approximate time of cell spreading. Bleb numbers per cell significantly decrease once the cell is spread (pink shaded area). **E**, Time lapse of a membrane tension measurement in a colony of ES cell. Time is in minutes:seconds. A red target is placed at the centre of the trap to help visualize bead displacement. **F**, Representative western blot for ERM, pERM and GAPDH in ES

cells and T24 cells and corresponding quantification (N=4, lines connect data points from individual experiments) **G**, Images of a single z-plane in fixed T16 cells. Three different cell morphologies are observed at T16: round, spread and blebbing. Cells were immunostained for pERM, and stained with DAPI and phalloidin. **H**, Similar as D with ERM instead of pERM. **I, J**, Estimated pERM and ERM and density in round, spread and blebbing T16 cells. Protein density was estimated from the mean intensity of sum z projections, corrected for background, and normalised to the mean of T16 round cells. (Means ± SD, N=3). For all panels, unless otherwise indicated, p-values established by Welch's unpaired student t-test.

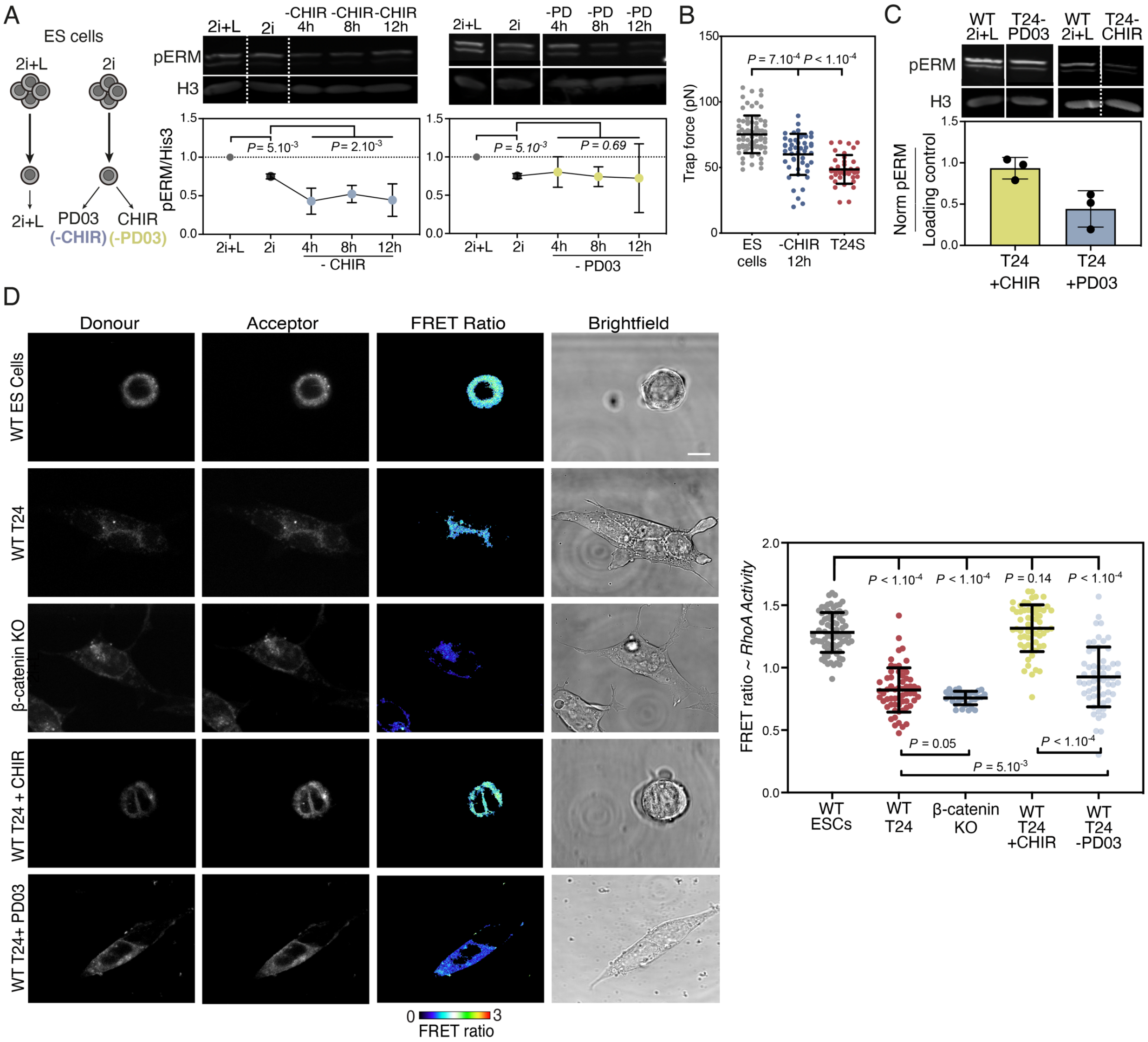

Figure S2: pERM activity and membrane tension during early differentiation are regulated by β-catenin and RhoA (related to Figure 2). A, Fluorescent Western Blot and associated quantification for pERM and Histone 3 (his 3) of cells cultured in 2i+L or from 2i and replated with PD03 or CHIR only for various timepoints (N=5). *P* values are calculated using a two-way analysis of variance (ANOVA), using experiment repeats and medium condition as variables, and indicated in the figure. **B**, Trap force measurements of WT ES, T24S (from Figure 1E) and cells cultured in medium lacking Chir (from 2i) (N=3). **C,** Top, representative fluorescent western blots for phospho-ERM and His3 of ES cells and T24 cells cultured in differentiation medium from 2i+L with either CHIR (annotated -PD03) or PD03 (annotated -CHIR). Bottom, associated quantification (N=3). **D**, Left, representative images of WT ES, T24, β-catenin KO cells and cells from 2i+L cultured for 24h in differentiation medium (N2B27) containing either PD03 (annotated -CHIR) or CHIR (annotated -PD03). Right, associated quantification (N=3). Graphical data represents mean ± SD. *P* values are calculated by Welch's unpaired student t-test (unless otherwise indicated). Scale bars = 10μm.

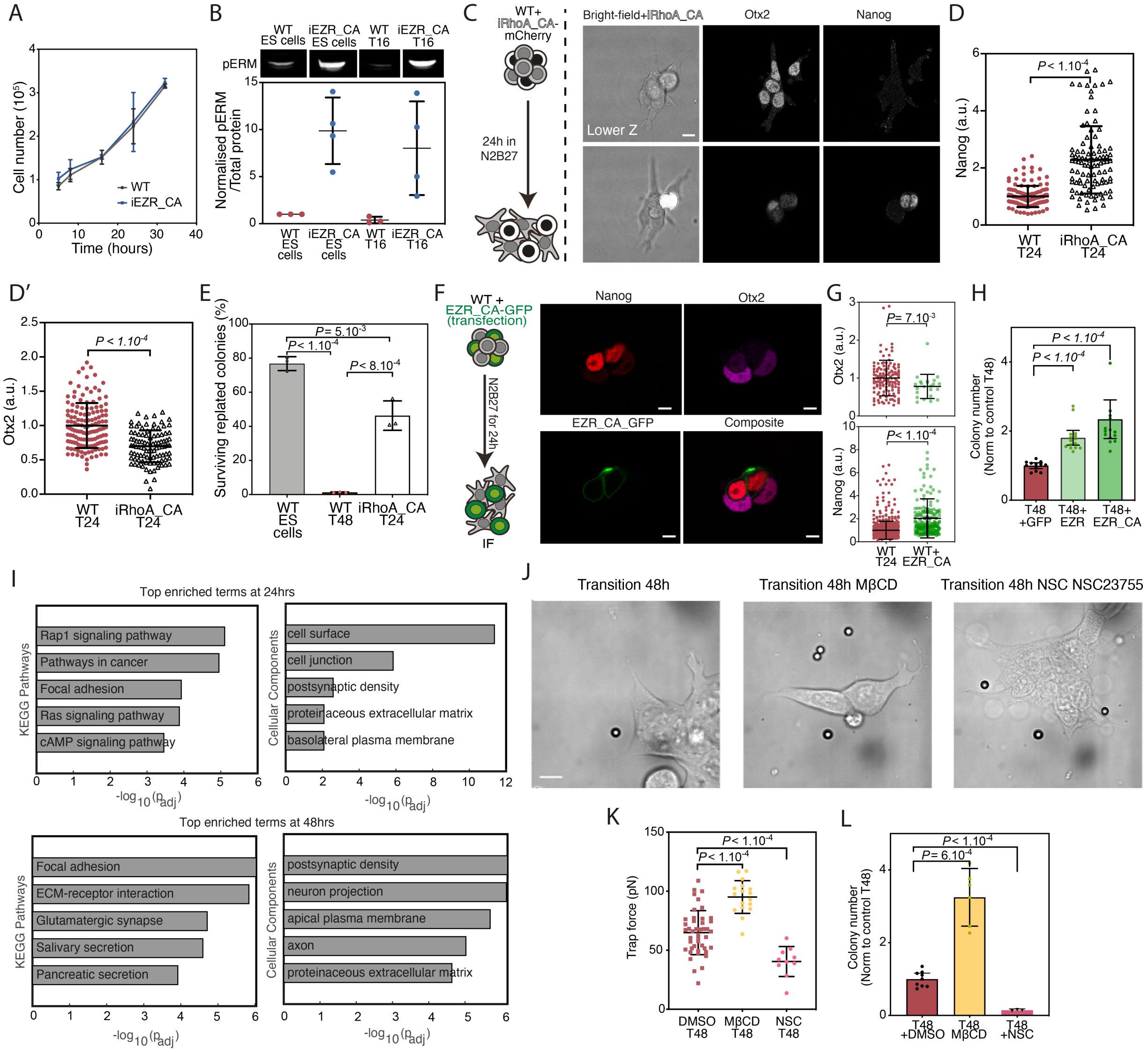

Figure S3: Increasing membrane tension inhibits early differentiation (related to Figure 3). A, Proliferation curves of WT and iEZR_CA ES cells (N=3). **B**, Quantification of phospho-ERM normalised to total protein levels in WT and iEZR_CA ES and T16 cells. Data are normalised to the mean of WT ES cells (N=4). **C,** Representative images of two z plane of a mix of WT and iRhoA_CA T24 cells immunostained for Otx2 and Nanog. **D,** Quantification of Nanog and Otx2 expression in WT and iRhoA_CA T24 cells, normalised to WT mean levels (N=3). **E**, Left, schematic of clonogenicity assay used as a measure of naïve pluripotency. Right: quantification of the percentage of surviving replated cells in a clonogenicity assay using WT and iRhoA_CA cells. Cells replated directly from 2i+L are used as a positive control (N=3). **F**, Representative single z-planes of a mix of control and EZR_CA transfected (positive for the EZR_CA _GFP) T24 cells immunostained for Otx2 and Nanog. **G**, Quantification of Nanog and Otx2 expression in T24 cells transfected or not with EZR_CA_GFP, normalised to T24 control mean

(N=2). **H,** Quantification of surviving replated cells in a clonogenicity assay using T48 cells transfected with either GFP (control), EZR, EZR_CA. (N=4) **I,** Top 5 KEGG pathways terms and Cellular Components terms that are enriched in the differentially regulated genes (n = 1165 genes at T24 and n = 1922 genes at T48). A threshold of > 5 was used for mean log2 expression, of >= 2.0 for fold enrichment, and of > 1% for proportion of regulated in each annotation. Adjusted p-value threshold was set at 0.1. The enriched annotations were sorted on increasing p-value and the top 5 were selected. **J**, Representative images of T48 cells treated with DMSO, NSC23766 or MβCD. MβCD was used at 1 mM for 1 h every 24 h. NSC23766 was used at 25 μM for 1 h every 24 h (N=3). **K**, Trap force measurements of T48 cells treated with DMSO, NSC23766 or MβCD at similar concentrations as in J. (N=2). **L**, Clonogenicity assay used to measure efficiency of exit from naïve pluripotency using T48 cells and different treatments to affect membrane tension. T48 cells replated from N2B27+DMSO were used as control for drug treatment. (N=3). Graphical data represents mean ± SD unless specified otherwise. Each condition is normalised to their respective control. *P* values are calculated by Welch's unpaired student t-test. Scale bars = 10μm.

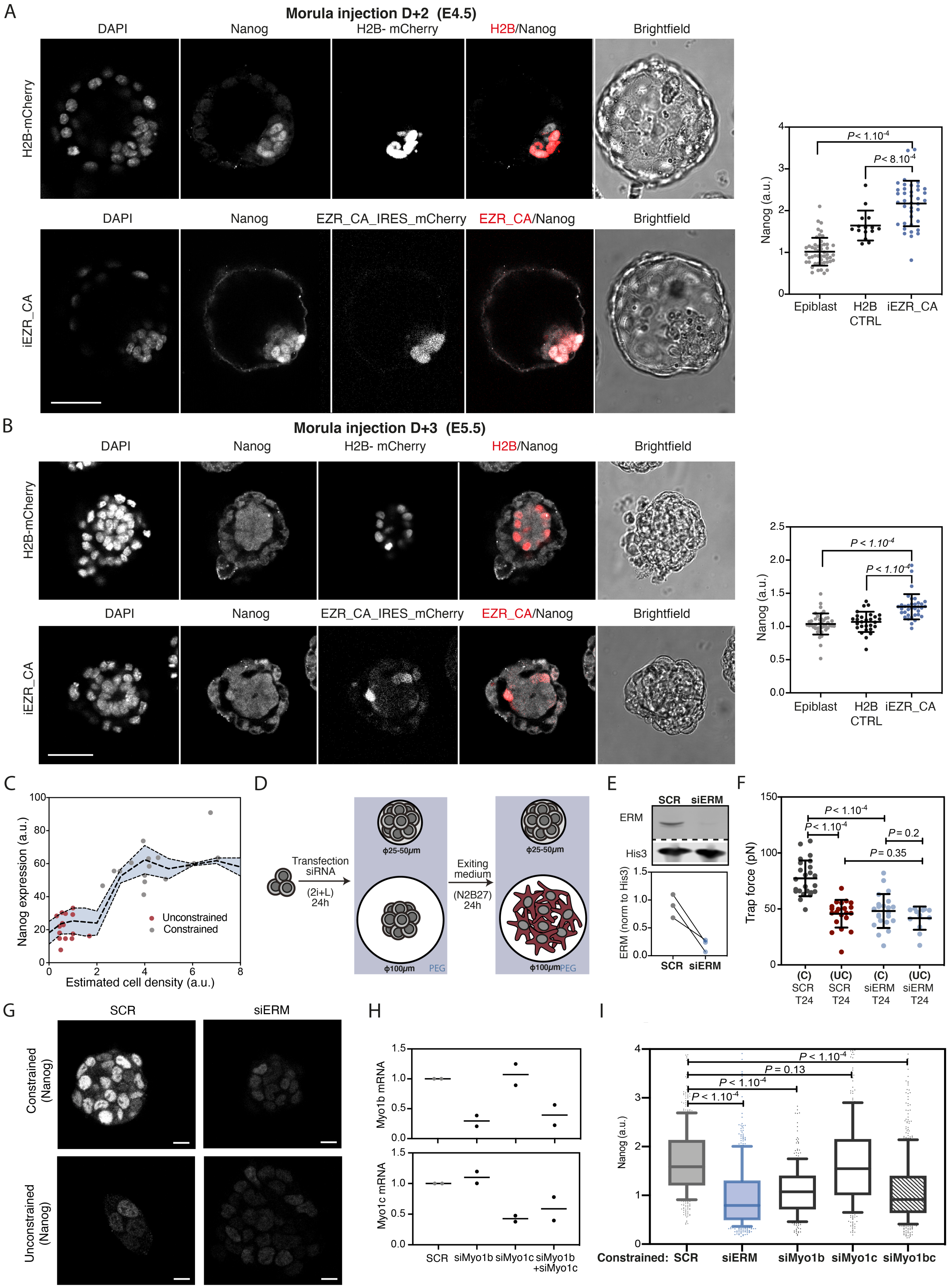

A
Morula injection D+2 (E4.5)
DAPI
Nanog
H2B- mCherry
H2B/Nanog
Brightfield
H2B-mCherry
EZR_CA_IRES_mCherry
EZR_CA/Nanog
iEZR_CA
Nanog (a.u.)
P< 1.10-4
P< 8.10-4
Epiblast
H2B CTRL
iEZR_CA
B
Morula injection D+3 (E5.5)
P < 1.10-4
C
Nanog expression (a.u.)
Estimated cell density (a.u.)
Unconstrained
Constrained
D
Transfection siRNA
(2i+L) 24h
ϕ25-50µm
ϕ100µm
PEG
Exiting medium
(N2B27) 24h
E
SCR
siERM
ERM
His3
ERM (norm to His3)
F
Trap force (pN)
P = 0.2
P = 0.35
(C) SCR T24
(UC) SCR T24
(C) siERM T24
(UC) siERM T24
G
Constrained (Nanog)
Unconstrained (Nanog)
H
Myo1b mRNA
Myo1c mRNA
SCR
siMyo1b
siMyo1c
siMyo1b +siMyo1c
I
Nanog (a.u.)
P < 1.10-4
P = 0.13
Constrained: SCR
siERM
siMyo1b
siMyo1c
siMyo1bc

Figure S4: Maintaining a high membrane tension impairs exit from naïve pluripotency independently of cell shape (related to Figure 4). A, B, Representative images and associated quantification of morula injection experiment in which either control cells (H2B-mCherry) or iEZR_CA were injected at the 8 cells stage into the mouse embryo, which was then maintained in culture for 48 or 72h (see methods for details) (N=2). **C**, Same Nanog expression as in Figure 4C plotted in function of estimated cell density. Cell density was estimated by counting the number of cells and dividing by the pattern area. Data are colored based on size of pattern (red for 100 µm diameter and grey for 25-50 µm diameter). **D**, Schematic of the micropatterning assay combined with transfection of plasmids or siRNA. **E**, Fluorescent Western blot (inverted contrast) for ERM and Histone 3 in cells transfected with siSCR and siERM showing the efficiency of the knockdown. **F,** Trap force measurement of cells cultured on micropatterns and transfected or not with siRNA against ERM proteins. (N=2). **G**, Representative images of single z-planes in fixed T24 cells treated with either SCR or ERM siRNA and cultured on micropatterns. **H**, Quantification of Myo1b and Myo1c RNA by qPCR assay in ES cells treated with either SCR, siMyo1b, siMyo1c and siMyo1b+siMyo1c. Data were normalised to Actb expression. **I**, Boxes and whiskers with 10-90 percentile plot of Nanog expression of T24 cells either transfected with siMyo1b, siMyo1c or siMyo1b+siMyo1c. Only data for cells constrained on small micropatterns are shown. *P* values are calculated by Mann-Whitney U here. Graphical data represents mean ± SD unless specified otherwise. *P* values are calculated by Welch's unpaired student t-test unless specidied otherwise. Scale bars = 10µm.

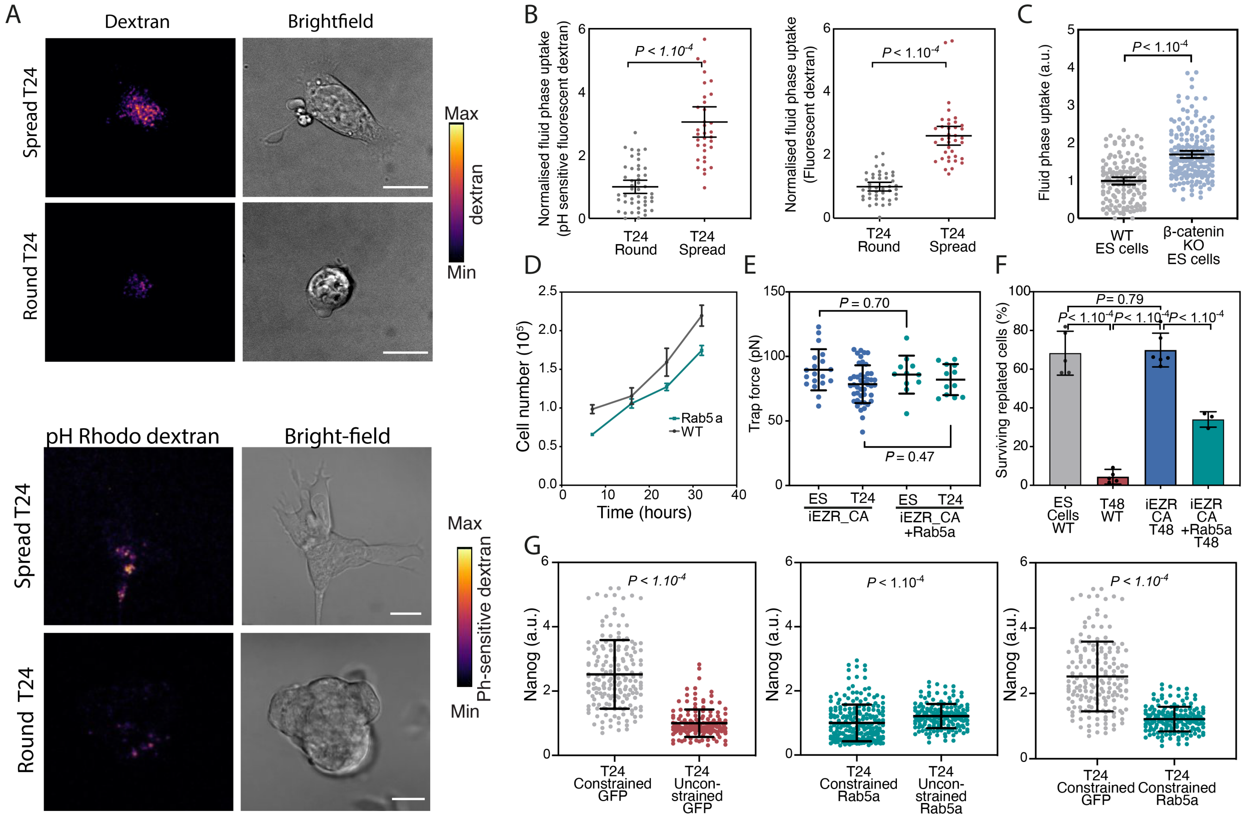

Figure S5: Endocytosis levels regulate exit from naïve pluripotency (related to Figure 5). **A**, Representative images of sum z-projections in round and spread T24 cells during a liquid phase uptake assay using fluorescent dextran or pH-sensitive fluorescent dextran. **B**, Left, quantification of liquid phase uptake using fluorescent dextran or pH-sensitive fluorescent dextran in T24 cells. Data are separated based on cell shape (round or spread). (Mean ± 95% Confidence Interval, N=2). **C,** Quantification of fluid phase uptake assay of WT & β-catenin KO ES cells. Data are normalised to WT ES cells average (N=3). **D**, Proliferation curves of WT and WT+Rab5a cells (N=2). **E**, Trap force measurements of iEZR_CA cells transfected with Rab5a, iEZR_CA trap force data are from Figure 3B. (N=2). **F**, Quantification of the percentage of surviving replated cells in a clonogenicity assay using ES and T48 cells, WT or iEZR_CA, transfected or not with Rab5a. Data for iEZR_CA T48 cells are from Figure 3E. (N=3).

G, Quantification of Nanog expression of fixed T24 cells transfected with GFP as control or Rab5a, cultured on micropatterns. This shows that Rab5a transfection can also rescue exit defects in constrained cells cultured on micropatterns. Data are normalised to cells cultured on 100 µm diameter patterns (Unconstrained) (*P* values in this panel are calculated by Mann-Whitney U test; N=3). Graphical data represents mean ± SD unless specified otherwise. *P* values are calculated by Welch's unpaired student t-test (unless specified otherwise) and are indicated in the figure. Scale bars = 10µm.

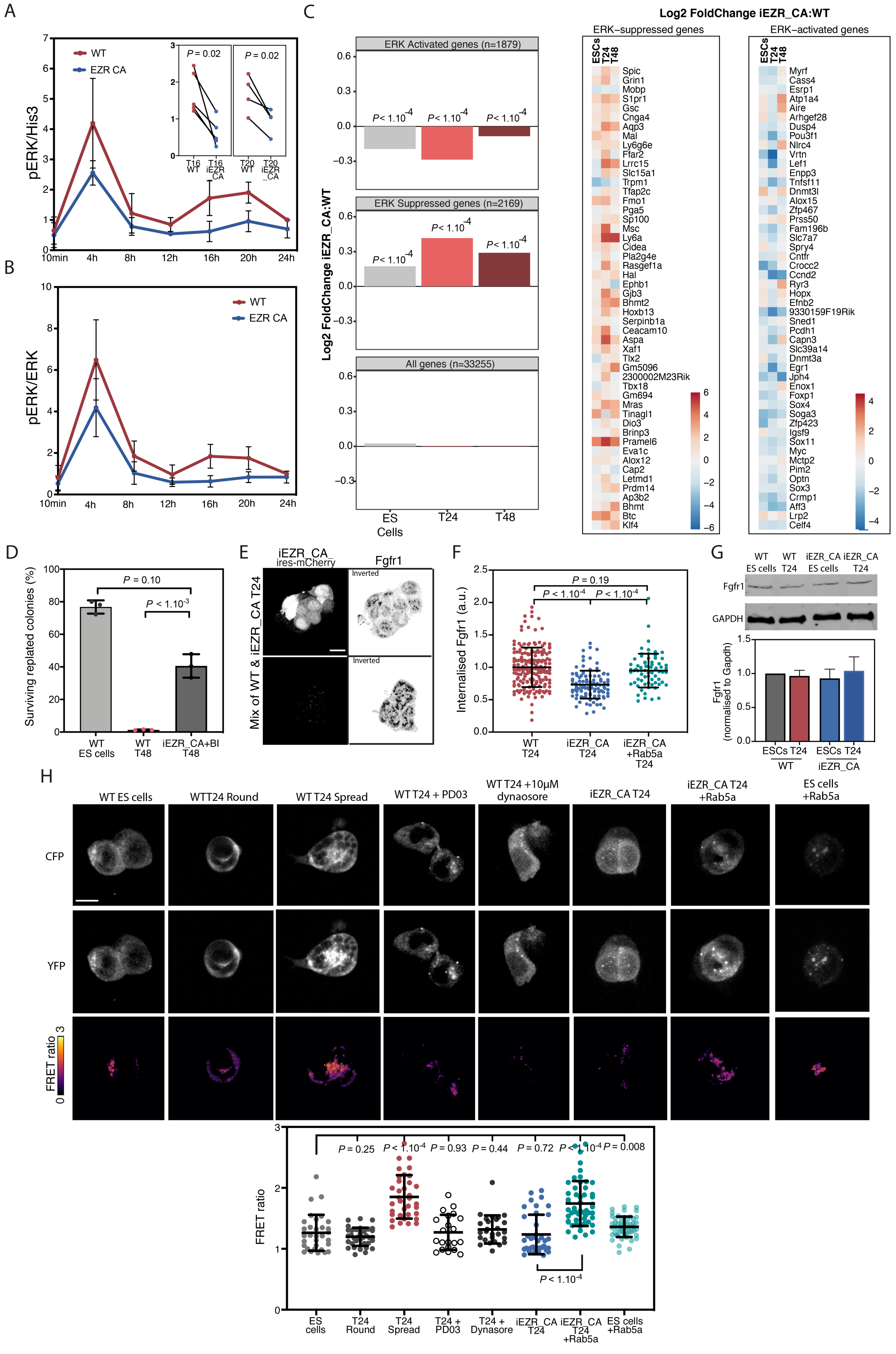
A
pERK/His3
WT
EZR CA
P = 0.02
T16 WT
T16 iEZR_CA
T20 WT
T20 iEZR_CA
10min 4h 8h 12h 16h 20h 24h
B
pERK/ERK
WT
EZR CA
C
Log2 FoldChange iEZR_CA:WT
ERK Activated genes (n=1879)
ERK Suppressed genes (n=2169)
All genes (n=33255)
P< 1.10^-4
ES Cells
T24
T48
Log2 FoldChange iEZR_CA:WT
ERK–suppressed genes
ERK–activated genes
ESCs
T24
T48
Spic
Grin1
Mobp
S1pr1
Gsc
Cnga4
Aqp3
Mal
Ly6g6e
Ffar2
Lrrc15
Slc15a1
Trpm1
Tfap2c
Fmo1
Pga5
Sp100
Msc
Ly6a
Cidea
Pla2g4e
Rasgef1a
Hal
Ephb1
Gjb3
Bhmt2
Hoxb13
Serpinb1a
Ceacam10
Aspa
Xaf1
Tlx2
Gm5096
2300002M23Rik
Tbx18
Gm694
Mras
Tinagl1
Dio3
Brinp3
Pramel6
Eva1c
Alox12
Cap2
Letmd1
Prdm14
Ap3b2
Bhmt
Btc
Klf4
Myrf
Cass4
Esrp1
Atp1a4
Aire
Arhgef28
Dusp4
Pou3f1
Nlrc4
Vrtn
Lef1
Enpp3
Tnfsf11
Dnmt3l
Alox15
Zfp467
Prss50
Fam196b
Slc7a7
Spry4
Cntfr
Crocc2
Ccnd2
Ryr3
Hopx
Efnb2
9330159F19Rik
Sned1
Pcdh1
Capn3
Slc39a14
Dnmt3a
Egr1
Jph4
Enox1
Foxp1
Sox4
Soga3
Zfp423
Igsf9
Sox11
Myc
Mctp2
Pim2
Optn
Sox3
Crmp1
Aff3
Lrp2
Celf4
D
Surviving replated colonies (%)
P = 0.10
P < 1.10^-3
WT ES cells
WT T48
iEZR_CA+BI T48
E
iEZR_CA_ ires-mCherry
Fgfr1
Inverted
Mix of WT & iEZR_CA T24
F
Internalised Fgfr1 (a.u.)
P = 0.19
P < 1.10^-4
WT T24
iEZR_CA T24
iEZR_CA +Rab5a T24
G
WT ES cells
WT T24
iEZR_CA ES cells
iEZR_CA T24
Fgfr1
GAPDH
Fgfr1 (normalised to Gapdh)
ESCs T24
WT
iEZR_CA
H
WT ES cells
WTT24 Round
WT T24 Spread
WT T24 + PD03
WT T24 +10μM dynaosore
iEZR_CA T24
iEZR_CA T24 +Rab5a
ES cells +Rab5a
CFP
YFP
FRET ratio
P = 0.25
P < 1.10^-4
P = 0.93
P = 0.44
P = 0.72
P = 0.008
ES cells
T24 Round
T24 Spread
T24 + PD03
T24 + Dynasore
iEZR_CA T24
iEZR_CA T24 +Rab5a
ES cells +Rab5a

Figure S6: ERK signalling and FGFR1 expression in exit from naïve pluripotency (related to Figure 6). **A**, Quantification of phospho-ERK normalised to Histone 3 levels across several fluorescent Western blots (including Figure 6A) done on WT and iEZR_CA cells at different timepoints during exit from naïve pluripotency. The first peak of ERK activation is likely a direct effect of the removal of the inhibitors. The timing of the second peak approximately coincides with the timing of the decrease in membrane tension and cell spreading (N=5). Inset, data for T16 and T20 cells where values for WT and iEZR_CA in individual experiments are connected by a line (N=5). **B**, Similar as panel a, for phospho-ERK normalised to total ERK levels. **C,** Left, bar plot showing the average log2 fold change over all ERK-activated (n = 1879) or ERK-suppressed (n = 2169) at each of the three time-points. P-values indicate if these averages are significantly different to averages over randomly selected genes (computed using permutation test with replacement). Right, heatmap of ERK-target genes, either suppressed or activated during exit from naïve pluripotency (see methods for details). Colormap indicates the log2 fold change between expression in iEZR_CA compared to wild-type cells. For both subsets (suppressed or activated), the top 50 genes are shown (sorted based on magnitude of Erk-regulation in wild-type cells at T24). **D,** Quantification of the percentage of surviving replated cells in a clonogenicity assay using WT T48 and iEZR_CA T48 cells treated with 3μM BI-D1870. Cells replated directly from 2i+L are used as a positive control (N=6). **E,** Representative single z-plane images of a mixed population of WT and iERC_CA (positive in the mCherry channel) T24 cells, immunostained for Fgfr1. **F**, Quantification of average intracellular Fgfr1 levels in T24 cells across different conditions (N=3). **G,** Fluorescent Western blot (inverted contrast) for Fgfr1 and GAPDH in WT and iEZR_CA ES and T24 cells, and corresponding quantification (data are normalised to GAPDH). (N=3). **H**, Top, representative images of z projections of fixed ES cells in different conditions. Bottom, associated quantification of FRET ratio for each condition. *P* values are calculated by Welch's unpaired student t-test using ES cells as reference. PD03 was used at 1 μM. Dynasore was used at 10 μM. Displayed pictures have been smoothed (Gaussian blur). *P*-values are calculated by Welch's unpaired student t-test (unless specified otherwise). Graphical data represents mean ± SD unless specified otherwise. Scale bars = 10 μm.
